# Removal of Pesticides from Waters by Adsorption: Comparison between Synthetic Zeolites and Mesoporous Silica Materials. A Review

**DOI:** 10.3390/ma14133532

**Published:** 2021-06-24

**Authors:** Magdalena Andrunik, Tomasz Bajda

**Affiliations:** Faculty of Geology, Geophysics and Environmental Protection, AGH University of Science and Technology, al. Mickiewicza 30, 30-059 Krakow, Poland; bajda@agh.edu.pl

**Keywords:** agricultural wastes, water treatment, sorption, organic compounds, silicates

## Abstract

Pesticides are pollutants found in wastewater due to increasing agricultural activities over the years. Inappropriate dosing of pesticides results in the dispersal of active ingredients in the environment. The complete removal of pesticides from wastewater is an immediate concern due to their high toxicity and mobility. At present, adsorption is one of the most widely used methods for pesticide removal, in which synthetic zeolites and mesoporous silica materials are extensively applied. This article presents a systematic and comparative review of the applications and comparison of these adsorbents, based on the data reported in the literature. The paper summarizes the information collected from various studies, including the type of adsorbents and pesticides used, experimental conditions, and results of each work. The studies analyzed were laboratory-based and show potential advantages for the treatment of pesticide-bearing waters using functionalized and unfunctionalized synthetic zeolites and mesoporous silica materials. As a whole, functionalized materials are reported to exhibit better removal performance for different pesticides than conventional materials. It is expected that the results of this review will help researchers to establish a powerful strategy for the abatement of pesticides in wastewater.

## 1. Introduction

Since the latter half of the nineteenth century, extensive agricultural use of plant protection products, which are referred to as “pesticides” (used henceforth in this paper), has been observed to pose a serious impact on soil, air, and water. As stated by the World Health Organization (WHO), the term “pesticide” is defined as any chemical compound that is used to kill pests (weeds, rodents, insects, fungi) [1]. The global demand for increased food production has not only led to significant deterioration of food quality, resulting in severe consequences on the environment but also caused public health issues due to overuse or misuse of pesticides [2,3]. It is assessed that more than 20% of the pesticides reach their nontarget species, as well as air, water, and soil [4]. Traces of these products are commonly detected in surface water, and more importantly, groundwater—a major source of drinking water on the world [5]. The presence of many types of pesticides and their derivatives in water is of great concern to the public and authorities, due to increased undesirable health effects resulted in the exposition on pesticides even at very low concentrations (pg/L to ng/L) [6].

Pesticides have greatly contributed to increasing agricultural yields by limiting pests and plant diseases and also by combating the insect-borne diseases in the human health sector [3,7]. For example, the production of food grains has increased dramatically in several countries since the implementation of pesticides. Although increases in productivity are attributed to different factors (e.g., use of fertilizers, better plants variations, and use of better machinery), pesticides have been an integral part of those processes by the reduction of losses from the weeds, diseases, and insect pests [8,9]. Furthermore, insecticides are the only way available to control the proliferation of deadly insect-borne diseases like malaria which results in an estimated 5000 deaths per day [10].

However, overuse and misuse of pesticides may have a negative impact on human health. Pesticides are used for controlling living species—they are biologically active substances that intervene with organisms and are characterized by different levels of toxicity [11]. These compounds are relatively stable and can bioaccumulate in living bodies. The toxicity of pesticides can be categorized as acute or chronic. Acute illness generally emerges after a short time of contact with the pesticide [12,13]. Suspected chronic effects resulting from regular exposure to small doses of certain pesticides may include birth defects, toxicity to fetus, genetic changes, blood, and nerve disorders. Furthermore, several studies have established a link between the exposure to pesticides and the frequency of chronic diseases that affect the nervous, reproductive, renal, cardiovascular, and respiratory systems in humans [14,15,16,17].

Based on their use and ability to kill organisms, pesticides can be classified as follows: insecticides, herbicides, rodenticides, fungicides, molluscicides, bactericides, avicides, virucides, algicides, acaricides, and miticides [9,18]. They can also be classified into four main groups according to the chemical nature of their active ingredients as follows: organochlorines, organophosphorus, carbamates, and pyrethrins and pyrethroids [19]. A brief description of those groups is presented in Table 1. In addition to the four main groups of pesticides mentioned above, there are few miscellaneous groups that are worth mentioning, such as phenoxyacetic acid (e.g., 2,4-D (2,4-dichlorophenoxyacetic acid) herbicide) or bipyridyls (e.g., paraquat and diquat herbicides). Inorganic pesticides are a minor category and include sulfur, copper, mercury, lead, and arsenic compounds. These pesticides are identified as extremely persistent and have caused serious problems of soil pollution in some areas; therefore, many of these are restricted [18,20,21].

**Figure 1:**
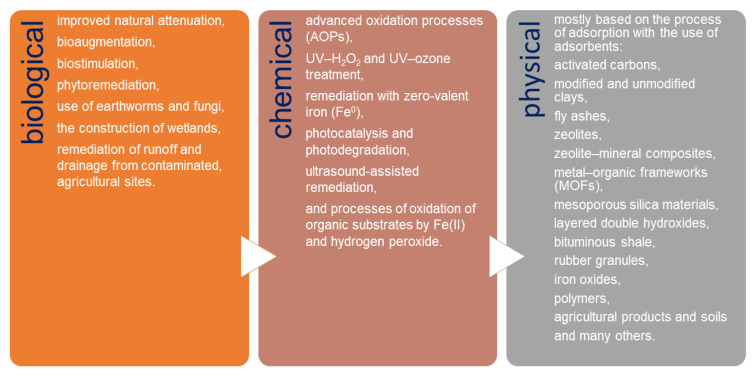
Methods used for pesticide remediation.

Currently, the removal of pesticides and their derivatives from the environment is one of the worldwide environmental alarms. Due to the wide use of different types of pesticides, it is extremely difficult to develop a single universal method for their removal. Basically, three groups of methods are used for pesticide remediation: biological, chemical, and physical (Figure 1). Biological remediation results in the transformation of organic compounds into harmless products such as CO_2_ and H_2_O [26,27,28,29,30,31,32]. These methods are of low cost and believed to be more environmental-friendly compared to the physical and chemical remediation methods. In chemical remediation, pesticides are converted into harmless compounds through certain agents by the way of chemical reactions [33,34,35,36,37,38,39,40,41]. Chemical treatment is usually combined with physical remediation processes; however, the costs of these combined treatments are very high and vary depending on the matrix [42]. Physical remediation is based mostly on the process of adsorption, which is one of the most commonly used methods for water purification because of its capacity, efficiency, and applicability on a large scale [3,22,43,44,45,46,47,48,49].

The present review compiles the data linked to the immobilization of pesticides on functionalized silica and aluminosilicate materials from aqueous solutions. Those materials with an ordered structure are gaining increasing interest in water treatment chemistry due to their noteworthy properties, including large pore size, high specific surface area, and abundant repertory of surface functional groups that can be tailored for selective adsorption of specific pollutants [50,51]. Zeolites and mesoporous silica materials are particularly in demand for aqueous separations involving large molecules due to the possibility of controlling pore diameter. Since porous silicates and aluminosilicates meet most of the criteria used for the selection of adsorbents, they have been extensively studied for the adsorption of inorganic and organic pollutants, and the adsorption efficiency for most of the studied contaminants is significantly high [52,53,54]. Thus, this review mainly aims to provide a summary of available information regarding the use of synthetic zeolites and mesoporous silica materials as adsorbents of pesticides. The readers are strongly encouraged to refer to the original research papers for detailed information. 

## 2. Characterization of Adsorbents

### 2.1. Mesoporous Silica Materials

Mesoporous silica materials are a new class of absorbents containing periodic arrays of channels and cavities [55]. The International Union of Pure and Applied Chemistry (IUPAC) defines them as materials with pore size in the range of 2–50 nm and exhibit an ordered arrangement of pores, which is responsible for their ordered structure [56,57]. The formation of those inorganic materials is based on the utilization of ordered surfactants as a template for the condensation of sodium silicate or silicon alkoxides around it [58]. Although the beginning of the synthesis of mesoscopic materials is dated back to the 1970s, mesoporous silica received attention only in the 1990s, when the Mobil Research and Development Corporation first synthesized mesoporous material from aluminosilicate gels with different morphological characteristics using the liquid-crystal template mechanism. Those materials were called Mobil Crystalline Materials or Mobil Composition of Matter (MCM) [56,57]. A few years later, silica nanoparticles with larger pores and thicker silica walls were produced at the University of California, Santa Barbara, which were named Santa Barbara Amorphous (SBA)-type materials [59]. Nowadays, there are various types of mesoporous silica materials available with different structural characteristics and functional groups [60].

The properties of mesoporous silica materials obtained depend strictly on the method used and the parameters of synthesis. These materials can be synthesized with different porosity, morphology, particle sizes, as well as with different functional groups, and hence, their physical and chemical properties can be tuned [61,62]. Usually, they have an enormous specific surface area (1000 m^2^/g or more), homogeneous pore distribution, and large volume and size of pores [63,64]. Generally, MCM-41 has a hexagonal structure with a pore diameter of 2.5–6 nm. MCM-48 has is cubic, whereas MCM-50 has a lamella-like arrangement [65]. SBA-type mesoporous silica materials differ from MCM type as they have larger pores with a size of 4.6–30 nm and thicker silica walls [66]. Based on the template used, SBA-based silica may be designated as SBA-11 (cubic), SBA-12 (three dimensional hexagonal), SBA-15 (hexagonal), and SBA-16 (cubic cage-structured) [56,67,68]. The structures of selected mesoporous silica materials are presented in the Figure S1.

Due to their diverse properties, mesoporous silica has a broad range of potential applications. In particular, they are used in medicine as, for example, delivery vehicles for pharmaceutical and biological molecules, host materials for bioimaging or biocatalytic agents, and platform materials for sensory or catalytic moieties [61,69,70,71]. They are also useful for the immobilization and separation of CO_2_, heavy metals, organic pollutants, volatile organic compounds, bioactive molecules, pigments, and dyes and thus play an important role in environmental protection [58,69,72,73,74]. Moreover, mesoporous silica materials attract a lot of academic interest because of their wide application in catalyst chemistry, electrochemistry, and energy storage [75,76,77,78].

### 2.2. Synthetic Zeolites

Zeolites include more than 50 aluminosilicate minerals with the general formula:

(1) $$M_{2/n}O•Al_{2}O_{3}•\mathrm{xSi}O_{2}•yH_{2}O$$

where M is any alkali or alkaline earth atom, n is the charge on that atom, x is a number varying from 2 to 10, and y is a number varying from 2 to 7 [79]. Zeolites have a three-dimensional crystalline structure made of AlO_4_ and SiO_4_. The connection of the atoms forces the structure of the zeolite—four oxygen atoms are located at the corners of each tetrahedron and are shared with the adjoining crystal tetrahedral, and each tetrahedron in the framework contains silicon or alumina as its central atom [79,80,81,82]. Such orientation of atoms results in the development of the structure full of pores and empty voids formed as cages and channels [83]. The crystalline lattice structure of zeolites is characterized by unique lattice stability and allows ion exchange as well as the accommodation of water molecules, new cations, and small organic molecules. Molecules and particles occurring in the voids and pores are loosely bound, and therefore, all the mentioned processes are reversible with no damage caused to the zeolitic framework [79]. However, this feasibility is determined by the crystalline structures and chemical composition and of a particular zeolite. The type of zeolites formed depends on the temperature, pH pressure, concentration of the reagent solutions, process of activation and aging period, and the contents of SiO_2_ and Al_2_O_3_ in the raw materials [80]. 

The most important zeolites physical properties are their bulk density, specific surface area, specific gravity, radius and volume of pores, which can correlate with the porosity of the materials, and cation exchange capacity (CEC) [84,85,86]. The CEC and adsorption properties, pH, and loss on acid immersion of zeolites are some of the chemical properties characteristic of zeolites and depend on the chemical composition of the zeolite. In general, for zeolites, a raise in the Si/Al ratio (from 0.5 to infinity) leads to changes in various parameters—acid resistivity, thermal stability, and increase in hydrophobicity, while hydrophilicity, acid-site density, and cation concentration may decrease [85,87]. The properties of synthetic zeolites are also influenced by the method used for synthesis and its related parameters. Both the surface area and CEC of zeolites exhibit substantial variations with a raise in alkali concentration and reaction time [84]. For example, the surface area tends to raise with an increase in the concentration of alkaline solution and the reaction time, while the CEC decreases with increasing concentration of alkaline solution; however, CEC also fluctuates arbitrarily with a raise in reaction time, which is usually ascribed to the changes in the size and volume of the pores [79]. The frameworks of selected zeolites are presented in the Figure S2.

The potential applications of zeolitic materials in industrial field depends on their type and properties. Zeolites (especially those with high CEC) can be applied in water purification. In particular, the use of zeolites has been extensively tested in solutions containing heavy metals and ammonium [88,89,90], which are commonly used as molecular sieves in gas purification technology [89,91]. Zeolites, as the most important solid catalysts, are used in traditional petrochemical industries, especially in cracking, isomerization, and hydrocarbon synthesis [92]. These materials are also used as detergent builders, as in contrast to conventional detergent builders, they are more environmentally friendly due to their ability to lower the hardness of water and insolubility [93]. Moreover, Canpolat et al. [94] showed that the use of zeolite as a replacement material in the production of cement increased the compressive strength of the obtained cement products. Besides these applications, zeolites play a significant role in many sustainable processes, especially in the fields associated with renewable energy and environmental protection, such as biomass conversion, fuel cell production, thermal energy storage, agriculture, or biomedicine [92,95,96].

## 3. Immobilization of Pesticides onto Different Materials

Table 2 summarizes the data presented in Section 3 and facilitates the navigation through the article.

### 3.1. Mesoporous Silica Materials

**Figure 2:**
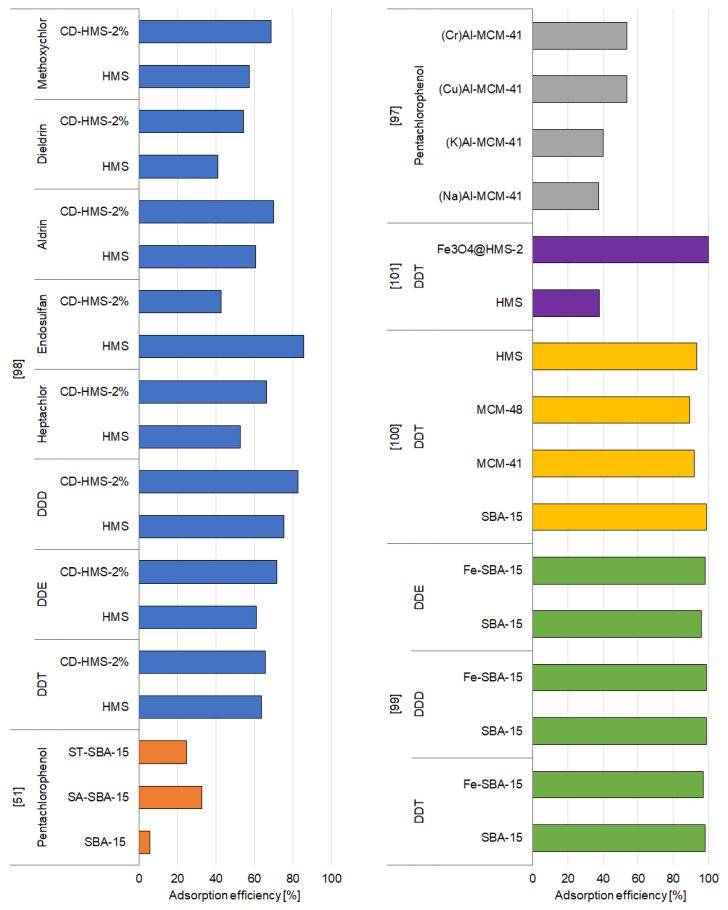
Effectiveness of removal of organochlorine pesticides by mesoporous silica materials.

**Figure 3:**
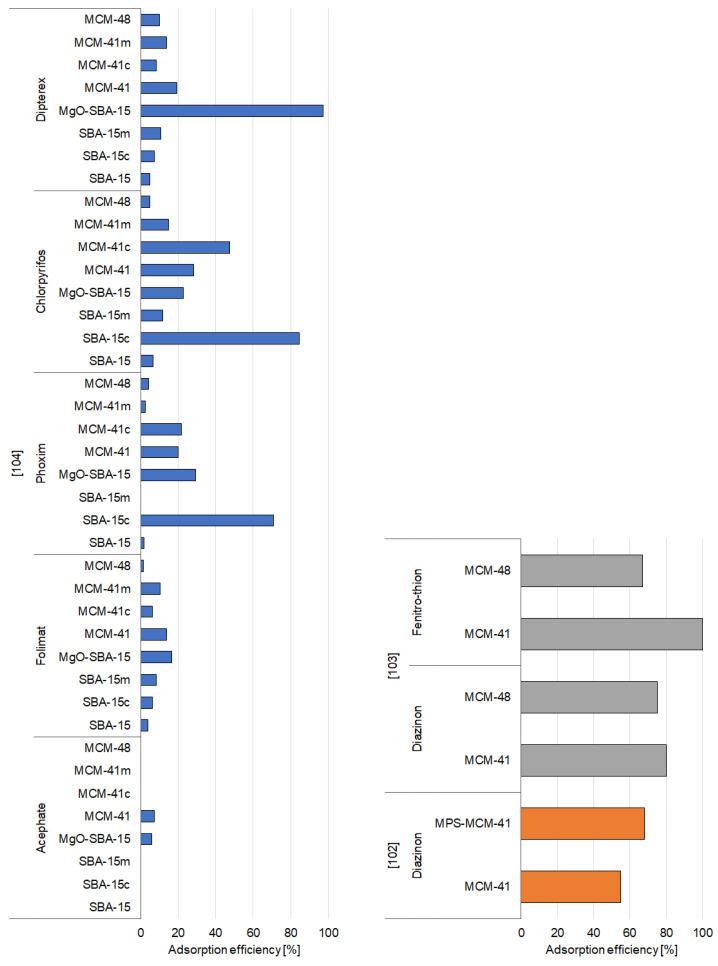
Effectiveness of removal of organophosphorus pesticides by mesoporous silica materials.

**Figure 4:**
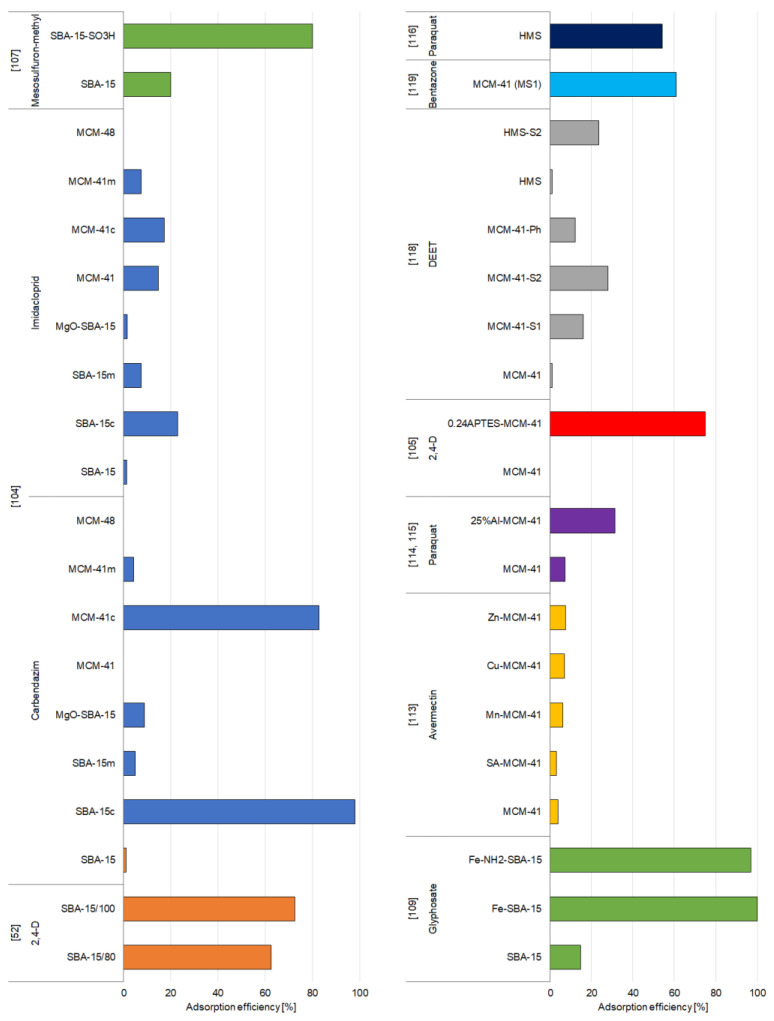
Effectiveness of removal of different groups of pesticides by mesoporous silica materials.

Functionalized and unfunctionalized mesoporous silica materials can effectively absorb different pesticides, as proven in various studies conducted during the period 2004–2020. The relative information obtained from these studies is given in Tables S1–S3 and Figure 2, Figure 3 and Figure 4, which summarizes the selected adsorption capacities of mesoporous silica-based adsorbents observed under the most efficient experimental conditions of pesticide removal. The structures of pesticides mentioned in the Section 3.1.1, Section 3.1.2 and Section 3.1.3 are presented in the Figures S3–S5.

#### 3.1.1. Organochlorine Pesticides

The results obtained by various scientists indicate that modification of mesoporous silica materials usually enhanced their adsorption capabilities that facilitated the removal of pesticides. Sawicki and Mercier [98] examined the influence of the amount of cyclodextrin (CD) used for the modification of HMS on its adsorption capacity for different pesticides. The synthesis of the adsorbents was based on the incorporation of different amounts of cyclodextrin into the pores, by the inoculation of the cyclodextrin groups into the channels of preformed mesoporous silica hosts, which resulted in varying amounts of binding sites [133]. The pesticides studied belonged to three chemical classes: hexachlorocyclohexane-based, hexachlorobicycloheptene-based, and *p*′,*p*-substituted biphenyl-based. The results of the study proved that unmodified HMS had a poor affinity toward all the tested groups of pesticides. Moreover, the amount of HMS used (1.4 or 7.1 g/L) had a negligible impact on the amount of pesticides adsorbed. Therefore, the authors concluded that adsorption or interaction between the adsorbent and adsorbate can be described as nonspecific, and results from the comparatively weak binding formed between the active sites on the surface and the pesticide. Incorporation of CD within the pore channels of HMS significantly improved its adsorption capacity at an amount of 2% (CD-HMS-2%) and 4% (CD-HMS-4%). In the case of functionalized materials (CD-HMS-6% and CD-HMS-8%), the adsorption efficiency was reduced. This may be because those materials had a significantly lower surface area and pore volume related to CD-HMS-2% and CD-HMS-4% due to increased congestion of the pore channels by the bulky cyclodextrin groups, which restricted the diffusion of the pesticide molecules. It was also indicated that functionalized HMS especially had a high affinity toward DDE, DDD, and DDT pesticides, which can be related to the geometry of the molecules. DDT and its derivatives consist of *p*-substituted phenyl groups which fit geometrically into the cavity of the cyclodextrin groups [98,134].

Due to the presence of numerous reactive sites, iron-loaded SBA-15 may also be considered as an efficient adsorbent of DDT (1,1,1-trichloro-2,2′bis(*p*-chlorophenyl)ethane) and its derivatives DDD and DDE (1-chloro-4-[2,2-dichloro-1-(4-chlorophenyl)ethyl]benzene and 1,1-dichloro-2,2-bis(4-chlorophenyl)ethene, respectively) [99]. The materials were synthesized by an in situ coating progress. The results of a study clearly showed that all samples of SBA-15 exhibited excellent adsorption efficiency toward the compounds DDD and DDE; however, the modification of SBA-15 influenced its adsorption properties. It was observed that the adsorption efficiency toward DDT slightly decreased with the surge of iron loading, DDD appeared to be modification-insensitive, and the removal of DDE increased after the modification of SBA-15. Compared with pure SBA-15, Fe-SBA-15 samples had reduced pore diameters, and thus, the authors suggested that pore diameter might have significantly influenced its ability to adsorb large organic molecules. These data are in agreement with the findings reported in the previously published work of Tian et al. [100]. Notwithstanding, the authors suggested that the increase in the adsorption efficiency of Fe-SBA-15 toward DDE might be related to the alkaline properties of the adsorbent [99].

Tian et al. [100] tested several different adsorbents against DDT—SBA-15, iron-modified SBA-15, MCM-48, MCM-41, and HMS (hollow mesoporous silica)—and noted that this pesticide was rapidly adsorbed. Within approximately 2 h, more than 50% of DDT was removed, while the equilibrium time was achieved within 6 h for SBA-15, 16 h for MCM-48, and 34 h for MCM-41 and HMS. The differences in the results were caused by the dissimilarities in the size of the pores and their connectivity. SBA-15 has a high density of OH^−^ groups on its surface and a larger pore diameter, which enable the diffusion of DDT molecules, and thus, for SBA-15 the equilibrium time of DDT was shorter. Moreover, SBA-15 has a smaller surface area, which indicates that surface area is not a key factor controlling the adsorption of DDT. MCM-48 generally exhibits the smallest nanopores; however, some of its nanopores have a larger diameter, and more importantly, the pores are connected with each other, which is in contrast to MCM-41 and HMS. This leads to a shorter equilibrium time for adsorption of DDT in the case of MCM-48 compared to MCM-41. The influence of the initial concentration of DDT was analyzed only for SBA-15, and the results indicated that the initial concentration did not significantly influence the adsorption efficiency [100].

In another study, Tian and his team [101] proposed a new concept for the synthesis of HMS with magnetic functionalization that can be applied for the immobilization of DDT. HMS was modified by encapsulating Fe_3_O_4_ nanocrystals, which were synthesized by a low-temperature solvothermal process, in mesoporous silica by following a packing approach, which resulted in the formation of core–shell-structured Fe_3_O_4_@HMS microspheres. The results obtained are in agreement with the study presented above [100]. Adsorption of DDT onto Fe_3_O_4_@HMS was very fast—almost 90% of DDT was adsorbed within 15 min. Afterward, the adsorption rate slowed down; however, the equilibrium was reached after approximately 1 h. On the other hand, unmodified HMS exhibited lower DDT adsorption efficiency compared to the iron-loaded HMS. The numerous reactive sites created by the formation of core–shell-structured Fe_3_O_4_@HMS microspheres combined with pores that are accessible for organic molecules and slight surfactant templates preserved in its pore channels improve the uptake affinity of organic molecules of Fe_3_O_4_@HMS [101,135].

Diagboya and coworkers [51] investigated the adsorption of pentachlorophenol using SBA-15-functionalized derivatives of aminopropyltriethoxysilane (SA-SBA-15) and tripolyphosphate (ST-SBA-15). In general, the organic modification was induced during the template-directed synthesis of the mesoporous material (SA sample), as well as post-synthetic modification (ST sample). The analysis of the adsorption rate revealed that equilibrium for the adsorbents was attained at 12 h. The authors connected the low adsorption on SBA-15 with the nature of its surface as it has no active functional groups to react with the predominant negatively charged pentachlorophenolate species [136]. Thus, the mechanisms behind the immobilization of pentachlorophenol by SBA-15 were identified as adsorption within mesopores by the charged pentachlorophenol molecules and very weak interactions with external wall surfaces. The situation is different in the case of SA-SBA-15, which has a positively charged amine group (NH_3_^+^) at a pH of 6.5. The removal of pesticide by this material was linked with the filling of mesopores and electrostatic interactions between the positively charged amine group of SA-SBA-15 and the anion of pentachlorophenol. The sorption of pentachlorophenol by ST-SBA-15 was higher compared to SBA-15. However, the considerably lower adsorption of ST-SBA-15 compared to SA-SBA-15 was related to the decrease in the amount of the NH_3_^+^ groups due to their interaction with tripolyphosphate ions [51].

Modification of MCM-41 with inorganic Na^+^, K^+^, Cu^2+^, or Cr^3+^ ions represents another way of mesoporous silica modification, and the adsorption of pentachlorophenol onto those adsorbents was reported to be promising [97]. MCM-41-based adsorbents were prepared by synthesizing the parent (Na)Al-MCM-41 first and then chemically modifying it by ion exchange, where Na^+^ ions were replaced by K^+^, Cu^2+^, or Cr^3+^. The resulting materials were named based on the rule (M)Al-MCM-41 (M = Na^+^, K^+^, Cu^2+^, Cr^3+^). Kinetic studies revealed that the adsorption of pentachlorophenol occurred rapidly—after 2 h, no changes were observed in its concentration. The impact of temperature on adsorption was analyzed at three different temperatures: 30, 40, and 50 °C. The results of the analysis showed that the adsorption of pentachlorophenol decreased with increasing temperature. The authors also tested the adsorption capacity of pentachlorophenol on (M)Al-MCM-41. The adsorption capacity of adsorbents decreased in the following order: (Cu)Al-MCM-41 > (Na)Al-MCM-41 > (Cr)Al-MCM-41 > (K)Al-MCM-41 (Figure 2). These results are consistent with the previous study of Khelifa et al. [137] who proved that the introduction of Cu^2+^ ions on the way of ion exchange in NaX zeolite debilitated the adsorbate–adsorbent interactions in favor of adsorbate–adsorbate interactions. The authors suggested that similar behavior could explain the high compatibility of (Cu)Al-MCM-41 toward pentachlorophenol, caused by the predominance of adsorbate–adsorbate interactions compared to adsorbate–(Cu)Al-MCM-41 interactions [97].

#### 3.1.2. Organophosphorus Pesticides

Diazinon is one of the most frequently analyzed pesticides in adsorption studies, due to its widespread use during the 1970s and the early 1980s for general-purpose gardening. In the study of Amani et al. [102], MCM-41 was synthesized via sol–gel process and further functionalized via a post synthesis grafting process with propyl methacrylate (MPS-MCM-41) to obtain an effective adsorbent. According to the results, the efficiency of removal of diazinon at low pH values was low; however, with the increase of pH from 5 to 9, the adsorption efficiencies ofMCM-41 and MPS-MCM-41 meaningfully raised. This was attributed to the increase of power in hydrogen bonding between the pesticide and the functional active sites on the surface of adsorbents. Initial concentration of diazinon in the solution also affected its adsorption efficiency. It was proven that at a low initial concentration (10 mg/L), the percentage of diazinon removal was 38% and 48.2%, while at 50 mg/L it was 52.4% and 66% for MCM-41 and MPS-MCM-41, respectively. Based on the results, the authors suggest that the occurrence of a specific functional group on the adsorbent’s surface implies the mechanisms of adsorption of diazinon. MCM-41 possesses hydroxyl functional groups, while the MPS-MCM-41 has both hydroxyl and propyl methacrylate functional groups. The hydroxyl groups can develop hydrogen bonds with the electronegative elements (N, O, and S) of the diazinon particle, while the propyl methacrylate groups can form hydrophobic interactions with diazinon’s methyl groups. It can explain the higher sorption of diazinon onto MPS-MCM-41 compared to pure MCM-41. For MPS-MCM-41, both hydrophobic interactions and hydrogen bonding can be suggested as the main mechanisms of adsorption, while for MCM-41 only hydrogen bonding may be recognized [102].

Adsorption of diazinon was also extensively examined by Armaghan and Amini [103]. They analyzed the removal of diazinon and fenitrothion onto MCM-41 and MCM-48. The adsorption of both compounds occurred very rapidly; in less than 5 min, most than half of fenitrothion was immobilized. The rate of adsorption of diazinon on MCM-41 was only subtly better than that on MCM-48, whereas the adsorption rate of fenitrothion on MCM-41 was notably better than that on MCM-48. Such effective adsorption was related to the high specific surface areas and pore size and structure of the two materials. After adsorption, the specific surface area of MCM-41 and MCM-48 decreased from 1030 and 1240 m^2^/g to 850 and 1100 m^2^/g for diazinon and 990 and 1210 m^2^/g for fenitrothion, respectively. This indicates that the pores and voids were occupied after adsorption. The decrease in surface area was caused by the filling of pores and channels. The most significant decline in surface area was noted for silica, which disruptively adsorbed the highest amount of pesticide. Therefore, the variances in removal capacity of diazinon and fenitrothion may be linked to their disruptive and nondisruptive adsorption. The authors indicated that the immobilization of fenitrothion, oppositely to diazinon, was not disruptive, and thus, fenitrothion may be remobilized with polar solvents [103].

The structure and morphology of absorbents appear to be important factors that influence the adsorption properties and can be managed during their synthesis. Various methods of synthesis were tested by Chen et al. [104], who studied the adsorption of seven different pesticides onto SBA-15, carbonized SBA-15 (SBA-15-c), SBA-15 monolith (SBA-15-m), MgO-modified SBA-15 (MgO-SBA-15), MCM-41, carbonized MCM-41 (MCM-41c), MCM-41 monolith (MCM-41m), and MCM-48. The removal efficiency of SBA-15 and MCM-48 for all the analyzed pesticides was found to be poor. Pure MCM-41 revealed better sorption capacity toward selected pesticides. This is because it has a greater surface area and different pores size than SBA-15. However, the removal efficiency was less than 30%. The MgO/SBA-15 sample immobilized 97% of dipterex, and its rate of immobilization was significantly greater than the pure SBA-15 and other samples (Figure 3). This indicates the involvement of alkali sites in the entrapping of organophosphorus pesticides. On the other hand, the removal efficiency for the other pesticides did not exceed 30%. Carbonization of SBA-15 (SBA-15c) caused a significant increase in the sorption of phoxim and chlorpyrifos. Similarly, the modification of MCM-41 with carbon nanoparticles improved the immobilization of chlorpyrifos, but the removal efficiency of the remaining pesticides was slightly decreased compared to MCM-41. The synthesized samples contained the template formed from channels that are occluded by micelles, and inside those channels, carbon particles may be developed [138]. SBA-15 monolith (SBA-15m) and MCM-41 monolith (MCM-41m) samples having a net-like structure to intercept targets did not show better adsorption properties. Their sorption efficiency was only slightly higher. This suggests that the morphological changes may have only a negligible role in capturing the organophosphorus pesticides. Moreover, the authors suggested that the capability of sorbents to trap pesticides was not strictly affected by their porosity and surface properties. MCM-48 and MCM-41m samples exhibited the largest surface area and regular pore structure, but their adsorption capacity was very low [104].

#### 3.1.3. Other Groups of Pesticides

Ganiyu et al. [107] used a modified version of mesoporous silica prepared by co-condensation method—propylsulfonic acid-functionalized SBA-15 (SBA-15-SO_3_H)—in their adsorption experiments. The results indicated that pure SBA-15, which has no sulfonic acid groups, showed a lower capacity of adsorption toward mesosulfuron-methyl than the acid-functionalized SBA-15-SO_3_H. The adsorption efficiency of SBA-15-SO_3_H increased significantly when the initial concentration of mesosulfuron-methyl was increased. This indicates that the propyl sulfonic groups were attached to the pore walls had a strong affinity for the pesticide. The kinetic study was conducted only for SBA-15-SO_3_H, and it was observed that equilibrium state was achieved after 30 h of reaction time with the adsorbate in solution; however, most of the pollutant was removed after 10 h [107].

The time taken for the adsorption of pesticides varies from a few minutes to a few hours and usually depends on the adsorbent used. A study showed that the adsorption of 2,4-D (2,4-dichlorophenoxyacetic acid) on SBA-15-templated mesoporous carbon occurred very quickly—most of the 2,4-D was adsorbed within a few minutes [52]. Briefly, adsorbents were synthesized by impregnation of the calcined SBA-15 samples with sucrose solution containing sulfuric acid, at different aging temperatures, which resulted in the development of inverse carbon replicas named carbon-SBA-15/80 and carbon-SBA-15/100. Adsorption kinetics showed that most of the pesticide was adsorbed in the first few minutes of the process. At lower pH values, the rate of removal by both adsorbents was considerably higher. For SBA-15/100, a dependence of removal on the solution pH was noted—at acidic pH values, the adsorption was nearly 100%, while at a pH higher than 5, the removal dropped to 50%. SBA-15/80 exhibited effective 2,4-D removal in a broad spectrum of pH values. The efficiency of removal varied between 100% in acidic pH and 80% in medium and alkaline pH (Table S3) [52].

Acidic conditions also appear to be better suitable for the adsorption of glyphosate (N-(phosphonomethyl)glycine). Rivoira et al. [109] devised adsorbents by encapsulating iron oxide nanoparticles within the porous-structured SBA-15 (Fe-SBA-15) and then functionalized the surface obtaining Fe-SBA-15 with (3-aminopropyl)triethoxysilane (Fe-NH_2_-SBA-15). The obtained results indicated that pure SBA-15 showed effective adsorption only at a low pH, while adsorption became insignificant at a neutral pH. The authors suggested that this decrease in adsorption efficiency could be caused by the repulsive ionic interactions preventing the removal, since at a higher pH, glyphosate is ionized, while SBA-15 surface is expected to become negatively charged [139]. At a low pH, partial retention was observed. This may be explained by hydrogen-bonding interactions occurring between the neutral form of glyphosate and the surface of SBA-15, whose surface charge is expected to be proximate to zero. In the case of Fe-SBA-15, the iron oxide provided new sites for the binding of the phosphate group in the structure of glyphosate on the way of development of monodentate and bidentate species [140]. The results observed at pH 2.1 support this hypothesis—the removal efficiency was significantly higher at this pH compared to pure SBA-15 (100%) (Figure 4). At pH 6, adsorption efficiency was lower (76%), mainly due to the change in surface charge, which at neutral pH becomes negative. The presence of amino groups in Fe-NH_2_-SBA-15 led to a opposite behavior. Adsorption efficiency was high only at acidic pH (97%), whereas at pH 6 the retention was only around 16%. This may be due to the presence of −NH_2_ moieties, which interact with glyphosate by anion-exchange interactions and hydrogen bonding. The anion-exchange interactions could be inhibited at pH 6 as the cationic sites are occupied with dissociated silanols [109].

Inorganic ions can also be applied for the functionalization of mesoporous silica materials [113]. A study analyzed Zn-, Cu-, and Mn-modified MCM-41 which were prepared as follows: mesoporous silica was first modified with salicylaldimine (SA-MCM-41) via co-condensation method, and the obtained product was further modified through coordination between metal ions and SA-MCM-41. Experiments with avermectin pesticide showed that adsorption capacities of the prepared adsorbents declined in the following order: Zn-MCM-41 > Cu-MCM-41 > Mn-MCM-41 > MCM-41 > SA-MCM-41 (Figure 4). The adsorption capacity of SA-MCM-41 was lower related to pure MCM-41, due to the blocking of pores after modification by salicylaldimine. However, further coordination with metal ions improved the interaction between avermectin and the mesoporous silica support. Metal ions were found to act as a bridge to coordinate with salicylaldimine-modified mesoporous silica and avermectin. Among the analyzed adsorbents, Zn-MCM-41 showed the highest adsorption capacity caused by its strongest coordination ability. Moreover, the authors concluded that modification with salicylaldimine and metal ions changed the kinetic adsorption mechanism from physical adsorption on the surface to intraparticle diffusion; inducing the importance of the pore effect on the adsorption process was significantly enhanced due to the favorable interaction between avermectin and the modified mesoporous silica [113].

Furthermore, similar studies performing modification of MCM-41 with inorganic cations proved that the modification of MCM-41 with Al^3+^ ions significantly improved its adsorption properties toward paraquat pesticide [114,115]. The adsorption of paraquat did not depend on its initial concentration in the solution. The authors suggested that up to some point the adsorption capacity showed a similar trend as the content of Al however only until the surface area remains relatively high. Incorporation of Al into the framework of mesoporous silica led to the generation of a negative charge, and therefore, cation that can balance the charge was needed. This cation may be able to exchange with pesticide, which occurs in the form of a dication. To prove that the higher the Al-loading, the better the sorption properties, the authors prepared four adsorbents with different concentrations of Al. Among them, 10%Al-MCM-41 showed the poorest adsorption efficiency, while 15%Al-MCM-41 and 20%Al-MCM-41 were comparable possibly due to their surface area and Al content which were not much different [114]. However, the adsorption capacity of 25%Al-MCM-41 was not found to differ from that of 15%Al-MCM-41 and 20%Al-MCM-41. It was probably caused by the fact that 25%Al-MCM-41 had the lowest surface area, despite the fact that the Al content was the highest, which indicates that a large amount of incorporated Al may not be suitable for improving the adsorption capacity of MCM-41 [114,115].

Similar to the modification of zeolites with inorganic ions, the incorporation of organic substances also significantly increases their adsorption capacity, as confirmed in the work of Ortiz Otalvaro and coworkers [105], who examined the influence of the addition of 3-aminopropyltriethoxysilane on the adsorption of MCM-41 toward pesticide 2,4-D. The modified MCM-41 (APTES-MCM-41) showed enhanced adsorption capacity for anions and reversed charge development, although no meaningful changes were exhibited in both the external texture and the mesoporosity. The adsorption of 2,4-D on the modified solid was much higher than on pure MCM-41. The results also indicated that the adsorption of 2,4-D strongly depends on the pH—low pH values were much more favorable. Expectedly, the immobilization of adsorbate was quick at the initial state (up to 5 min), which may be connected to the direct binding between the adsorbate and aminopropyl surface groups of adsorbent; however, further adsorption was much slower (above 5 min). This can be due to the fact that 2,4-D requires a more time to occupy all the binding sites due to the narrow pores. The authors indicate that the main factors responsible for the adsorption reactions were electrostatic attractions combined with hydrogen bonds between the pesticide’s functional groups and the silica’s amino groups [105].

Moreover, Trouve et al. [118] proved that modifications allow adjusting the hydrophilic/hydrophobic equilibrium of the surface of mesoporous silica, which is an effective way to improve its ability to adsorb organic pollutants. They used functionalized MCM-41 and HMS as adsorbents for the pesticide N,N-diethyl-m-toluamide (DEET). The adsorbents were prepared by metal-free synthesis of MCM-41 and HMS, which were further functionalized by silylation of the superficial silanol groups. The obtained, silylated MCM-41 and HMS samples were labeled as MCM-41-Sx (x = 1 or 2) and HMS-Sx (x = 1 or 2). The modified MCM-41 sample was also subject to a second round of functionalization based on the co-condensation of silica precursors (MCM-41-Ph) and phenyl. The experiments of adsorption revealed that regardless of the type of silica, the unmodified adsorbents exhibited a low DEET adsorption efficiency. This was due to the fact that these samples exhibited a significant silanol density in the pores, which induced preferential adsorption of water rather than organic pollutants. All silylated silica samples adsorbed much higher amounts of DEET. The presence of phenyl groups also significantly improved the adsorption properties of MCM-41-Ph related to pure MCM-41; however, the amount of DEET absorbed was lower compared to the silylated samples. Based on these findings, it was suggested that the maximal quantity of DEET that can be immobilized by the silylated MCM-41 and HMS was about 180 mg/g, which coincides to approximately 50% occupancy of the porous volume by pesticide (Table S3). This is associated with the hydrophobization of the silica surface. The reduction of the capillary water condensation in the pores enhanced the immobilization of DEET by the silylated samples. The increase was more than 20-fold compared to the corresponding unmodified silica. The authors, therefore, suggested that balancing the hydrophilic/hydrophobic properties of mesoporous materials through appropriate hydrophobic derivatization is the main variable for increasing their adsorption capacities [118].

However, modification is not always necessary to achieve satisfactory adsorption—some pesticides may also be effectively immobilized by pure, unmodified mesoporous silica-based sorbents. Bruzzoniti et al. [119] studied the adsorption of bentazone onto pure MCM-41 (in their work, it was referred to as MS1). The data obtained indicated that bentazone removal from the solution was very rapid—most of the adsorbate was immobilized within the first few minutes of contact. After 6 h of contact, the amount of adsorbed pesticide decreased, probably due to desorption processes. It may be attributed to the competitive sorption of water particles on MCM-41 active sites by developing of H-bonding with the silanol groups on the silica surface [141]. The reaction of the surface of the adsorbent with an aqueous solution may lead to hydrophilization of the surface on the way of hydration and the development of surface hydroxyls. The experiments analyzing the influence of initial bentazone concentration revealed that the level of immobilized bentazone increased with increasing equilibrium concentration; however, 100% removal was never reached, even at low concentrations. This confirmed that sorption is an equilibrium process, and that full load was not attained in such conditions. The affinity toward bentazone was also found to be strongly influenced by pH (2–7). Acidic pH values enhanced the retention of bentazone on MCM-41, whereas at neutral pH values, the retention was equal to zero. The reduction of adsorption with raising pH implies that hydrogen bonding and ionic interactions have a significant impact on the pollutant–sorbent interactions. At low pH, the form of bentazone is undissociated and silica has a surface charge close to zero, whereas at a higher pH bentazone occurs in the ionized form, and the charge of the silica surface becomes negative. Thus, the repulsion between the pesticide molecules and the surface of the silica decreases the removal efficiency [119].

Pesticides that occur in the environment in cationic form can also be effectively immobilized by pure, unmodified HMS mesoporous silica, which was hydrothermally synthesized [116]. In a study, experiments of the adsorption of dicationic herbicide paraquat onto HMS revealed that paraquat adsorption is very fast and occurs between 0 and 5 min. Above 5 min, the adsorption was much slower, while equilibrium was reached after 50 min. The authors predicted that paraquat ions could bind directly with the active centers of silica and the negatively charged silanol groups in the pores [142]. However, there is no unanimity related to the nature of interactions that are responsible for the slower adsorption (above 5 min of adsorption). This can be connected with the adsorption equilibrium that is attained at the point when the speed of adsorption and desorption resemble each other. Moreover, some other phenomena may occur like diffusion into pores, intraparticle diffusion, or surface binding heterogeneity [143]. Furthermore, the adsorption capacity depends on pH. The process is considerably fast at pH 9.5 and its speed declines meaningfully at pH 7 and 4.4. It is proved that at pH above 4.4, the surface of HMS is negatively charged mainly because of the changeable charge from the pH-dependent surface hydroxyl sites, and thus with the increase of pH, the amount of available bonding sites would also increase. The adsorption of paraquat also is dependent on the temperature and decreases as the temperature raises from 5 to 45 °C. This could be due to the development of outer-sphere complexes or ionic pairs, which competes with the cations of the supporting electrolyte [116].

In order to examine adsorption possibilities of immobilization of carbendazim and imidacloprid, various methods of synthesis of mesoporous silica materials were tested by Chen et al. [104]. They used followed adsorbents: SBA-15, carbonized SBA-15 (SBA-15-c), SBA-15 monolith (SBA-15-m), MgO-modified SBA-15 (MgO-SBA-15), MCM-41, carbonized MCM-41 (MCM-41c), MCM-41 monolith (MCM-41m), and MCM-48. The removal efficiency of pure SBA-15, MCM-41, and MCM-48 for the analyzed pesticides poor, caused possibly by the improper geometric shape, which is ineffective in entrapping the pesticide (Table S3). Preparation of sorbents with net-like structure to intercept targets (SBA-15m and MCM-41m) also did not enhance their adsorption properties, which suggests, that changes in morphology did not influence immobilization of pesticides. However, carbonization of SBA-15 (SBA-15c) and MCM-41 (MCM-41c) caused an adsorption increase, especially for carbendazim, due to formation of numerous carbon particles inside the channels, which helps entrapping pesticides [104]. 

### 3.2. Synthetic Zeolites

**Figure 5:**
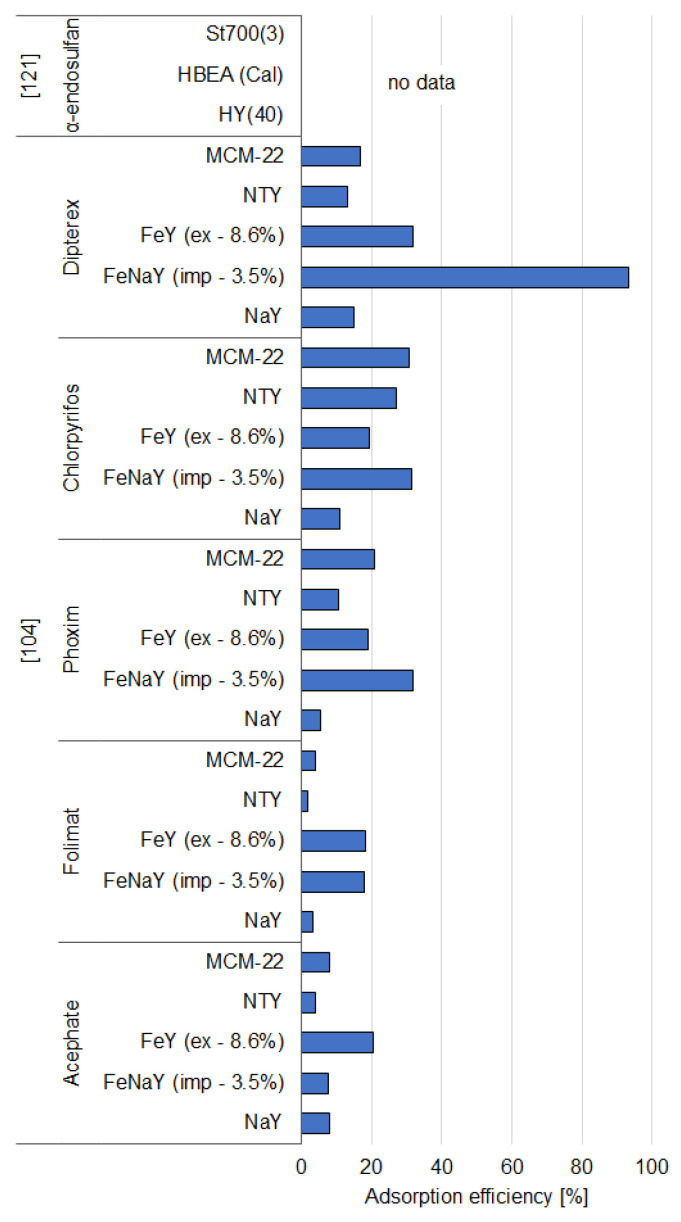
Effectiveness of removal of organochlorine and organophosphorus pesticides by synthetic zeolites.

**Figure 6:**
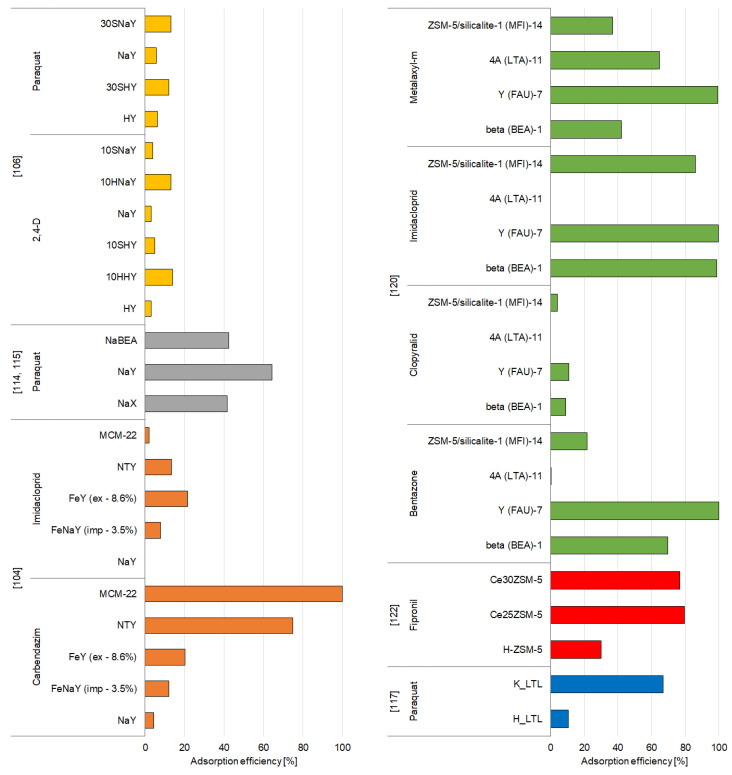
Effectiveness of removal of different groups of pesticides by synthetic zeolites.

**Figure 7:**
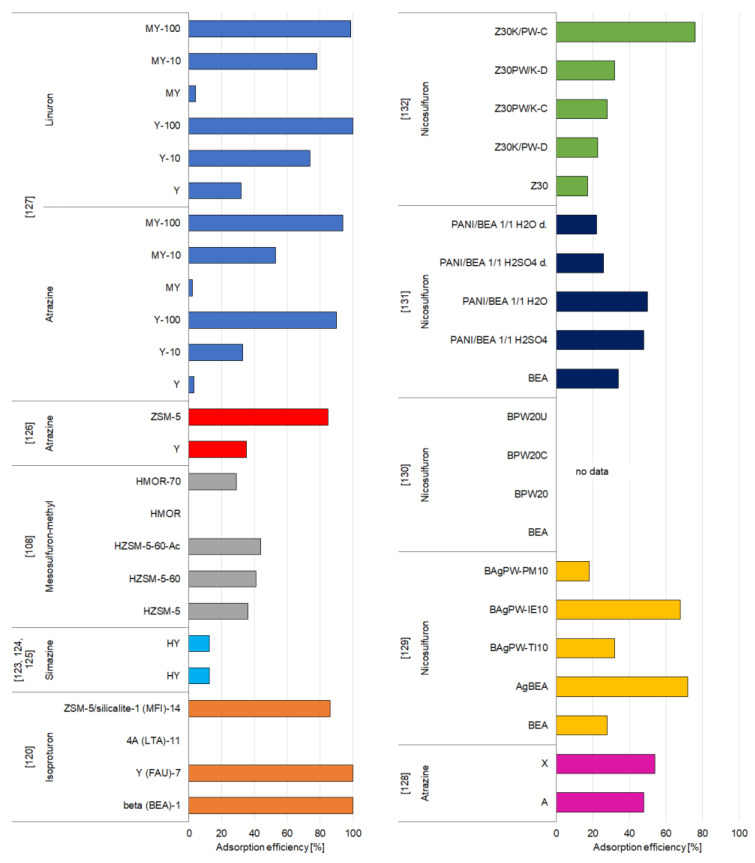
Effectiveness of removal of triazine and urea pesticides by synthetic zeolites.

**Figure 8:**
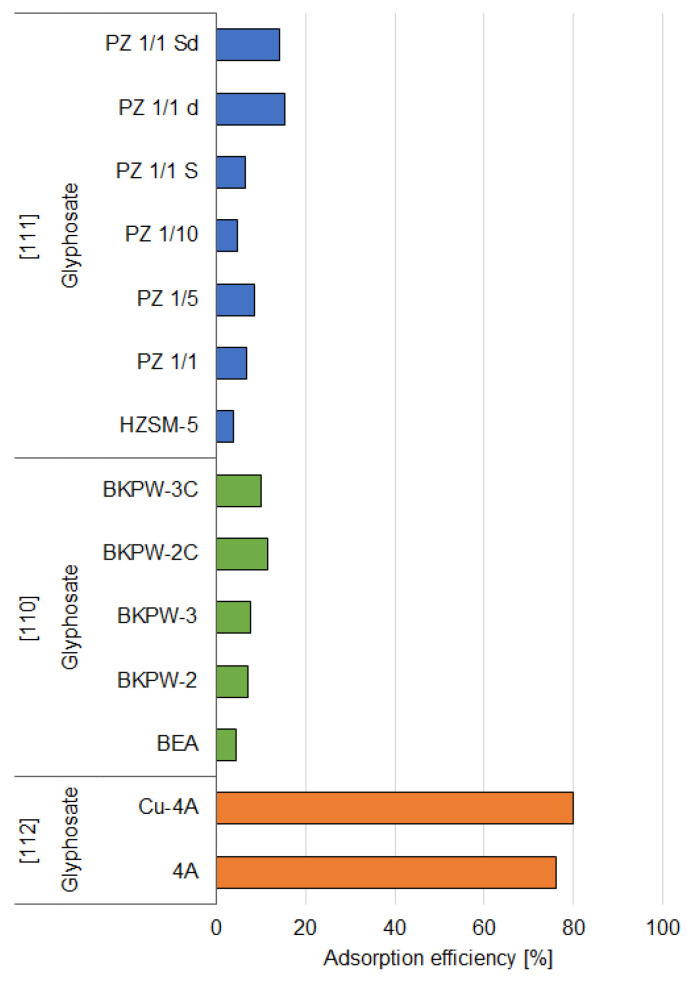
Effectiveness of removal of glyphosate-based pesticides by synthetic zeolites.

Generally, both modified and unmodified synthetic zeolites are well-recognized adsorbents, as they are proven to be efficient in adsorbing various organic and inorganic pollutants. Their extensive surface area and high pore volume result in their efficiency in immobilization of pesticides, which has also been proven by many studies conducted in 1994–2020. Tables S4–S7 and Figure 5, Figure 6, Figure 7 and Figure 8 present a summary of the research activities and the related data concerning the adsorption by zeolite-based adsorbents under the most efficient experimental conditions of pesticide removal. The structures of pesticides mentioned in the Section 3.2.1 and Section 3.2.2 are presented in the Figures S3–S5.

#### 3.2.1. Organochlorine and Organophosphorus Pesticides

Yonli and coworkers [121] also investigated the use of HY zeolites and steamed HBEA zeolites as adsorbents of α-endosulfan from water. They used faujasite zeolite samples (HY) as adsorbents without any posttreatment. The HBEA zeolites were dealuminated by a steaming procedure to eliminate the framework Al atoms along with the Al-OH groups. The obtained materials were labeled as St500(3), St600(3), and St700(3), depending on the temperature of steaming. The results of the experiments with these zeolite samples showed that their removal efficiency raised when their Brønsted acidity decreased, and the changes were more meaningful for the HY zeolite than for the HBEA zeolite. This suggests that the Brønsted acid sites may be disfavored for the α-endosulfan. What is more, during the removal of these sites, hydrophobicity increases, which is caused by the limited adsorption of water. The removal of α-endosulfan is promoted by the hydrophobization of the adsorbent; however, due to the size of the pores, the HY samples were more affected. The HY(40) zeolite was thus identified as the most effective adsorbent because of the lower acidity and immobilization of pesticide molecules on the Brønsted acid sites and the concessional adsorption of water molecules in the mesopores. On the contrary, for HBEA zeolite, the immobilization of α-endosulfan on Brønsted sites was limited by the favorable adsorption of water on these sites [121].

The adsorption of five organophosphorus pesticides onto unmodified and modified zeolites was studied by Chen et al. [104]. They obtained FeNaY sample by impregnating NaY zeolite with 3.5% of Fe. FeY zeolite was developed by using ion exchange, and the NTY sample was dealuminated to remove the extra Al species outside the framework. The results showed that MCM-22 zeolite is not able to effectively immobilize organophosphorus pesticides. NaY zeolite has a considerably large pore size and the super cage structure, although it did not efficiently adsorb the analyzed pesticides, mostly due to its hydrophilicity. Dealumination (sample NTY) caused a reduction in hydrophilicity and increased the Si/Al ratio, which resulted in higher adsorption capacity. Similarly, the sample impregnated with Fe (FeNaY) captured more that 90% of dipterex; however, the ion-exchanged sample (FeY) exhibited poor adsorption capacity toward pesticides (Figure 5). Moreover, the authors indicated that the capability of entrapping pesticides did not simply relate to their porosity and specific surface area; however, the large microporous surface area seemed to be beneficial, since dealuminated and Fe-impregnated zeolites (NYT and FeNaY, respectively) exhibited relatively higher adsorption [104].

#### 3.2.2. Other Groups of Pesticides

Modification of zeolites with organic and inorganic compounds is one of the most effective ways to improve their adsorption properties. Zeolite HY, which is characterized by a high Si/Al mole ratio of 100, has been used as a parent zeolite for different modifications: HHY—HDTMA-modified zeolite Y in H-form, SHY—SDS-modified zeolite Y in H-form, NaY—zeolite Y in Na-form, HNaY—HDTMA-modified zeolite Y in Na-form, and SNaY—SDS-modified zeolite Y in Na-form [106]. All these adsorbents have been tested against paraquat and 2,4-D. The functionalization of HY and NaY zeolites with different amounts of HDTMA or SDS (hexadecyltrimethylammonium chloride and sodium dodecyl sulfate, respectively) and the formation of admicelles on the surface of surfactant zeolite influence the adsorption ability of zeolite toward 2,4-D and paraquat. Modification of zeolite with SDS surfactant was found to increase the adsorption of paraquat; however, the high amounts of HDTMA caused a decline adsorption efficiency. For 2,4-D, zeolites were more efficient while modified with a greater amount of HDTMA and SDS. Moreover, for SDS-modified zeolites, the presence of water and the method of adsorbent preparation affected the pesticide adsorption capacity. SDS-modified zeolites exhibited higher adsorption efficiency toward 2,4-D when they were heated in at 110 °C for 3 h; however, for removal of paraquat, the adsorbents dried in a desiccator for 3–5 days were more effective. During quick-drying in the oven, poorly bounded water molecules are eliminated, and highly polarized molecules remained attached to negatively charged groups on SDS, which provide reactive sites for Na^+^ and enable bonding between Na and the 2,4-D in anionic form. On the other hand, drying in a desiccator allows retaining the clusters of water particles around the negatively charged head groups of SDS, which weakens the reactions between the SDS and sodium ions and thus enhances the ion exchange between the Na ions on the adsorbent surface and the paraquat ions. The adsorption characteristics of adsorbents also depend on the pH of the solution. Raising the pH of the solution decreased the capacity to adsorb 2,4-D but raised the adsorption efficiency of paraquat. All materials exhibited higher adsorption efficiency toward paraquat with increasing pH, with the highest efficiency at pH 11, while for 2,4-D pesticide, the adsorption was significantly higher at low pH (pH 3), mainly due to the competition between OH^−^ and 2,4-D anions at high pH values [106].

Paraquat herbicide was also studied by Rongchapo and coworkers, who utilized NaY and NaBEA zeolites [115] and NaX zeolite [114] derived from rice husk silica as adsorbents. The adsorption efficiency of NaY was greater than that of NaBEA. The authors suggested that adsorption capacity showed a similar trend as the Al content. The Si/Al ratio in NaBEA is usually 8–20, while in NaY the ratio is 1.5–3.0. The size of paraquat molecule (0.64 × 0.34 × 1.34 nm) is smaller than the pore sizes of NaY and NaBEA, which indicates that adsorption occurs by ion exchange. Al atoms present on the surface of zeolite induce the generation of a negative charge, which can be balanced by paraquat cations [115]. The amount of paraquat adsorbed onto NaX zeolite is similar to that of NaBEA, and much less compared to adsorption onto NaY zeolite [114,115]. Both zeolites have the same framework type (FAU) and similar pore size but differ in the surface area and Si/Al mole ratio. The Si/Al ratio of zeolite NaX (1.27) is significantly lower than NaBEA (14.2) and even NaY (2.2). However, the surface area of NaX (735 m^2^/g) also differs from that of NaY (870 m^2^/g) and NaBEA (632 m^2^/g), which suggests that the surface area may also be a key factor controlling the adsorption capacity of zeolites [114,115].

The importance of the Si/Al ratio with regarding to the adsorption capacity of zeolites was also emphasized by Insuwan and Rangsriwatananon [117], who studied zeolite LTL modified with potassium (K_LTL) and in proton form (H_LTL) as adsorbents for paraquat. The results of their study revealed that the tendency of adsorption efficiency was comparable to that of aluminum content, which suggests that the immobilization depends on the Si/Al ratio. K_LTL has a lower Si/Al ratio and thus a greater exchange capacity than H_LTL. H_LTL was synthesized by changing K_LTL into NH_4_LTL by ion exchange. This resulted in the deammoniation and development of Brønsted acidic bridging protons in the framework. Aluminum was eliminated, and silicon atoms moved to occupy the unbonded sites. Furthermore, both zeolites exhibit various ionic sizes and various affinities for the negatively charged sites. The K^+^ ions may form electrostatic attraction forces, while the hydrogen ions are usually bounded on the way of H bonding. The H bonding is very strong at the negatively charged sites, and thus, zeolite H_LTL is barely replaced with paraquat, compared to K_LTL [117].

One of the main mechanisms of pesticide immobilization is the electrostatic interactions between the functional groups of the adsorbent and the pesticide molecules. To effectively immobilize the pesticides in cationic forms, adsorbents with a lot of negatively charged adsorption sites are required. Cerium-modified HZSM-5 zeolite is an example of such adsorbent [122]. The HZSM-5 zeolite was functionalized with ceric ammonium nitrate salt using 5%, 10%, 15%, 20%, 25%, and 30% cerium nitrate solution by applying the refluxing method, and the samples obtained were designated as Ce5ZSM-5, Ce10ZSM-5, Ce15ZSM-5, Ce20ZSM-5, Ce25ZSM-5, and Ce30ZSM-5, respectively. The results observed indicated that the modification of zeolite enhanced its adsorption properties, and the sample Ce25ZSM-5 was the most efficient in the removal of fipronil (Figure 6). The replacement of positive H or Na ion with a polyvalent cerium cation stabilized the adsorbent structure by raising the amount of reactive sites. Adsorption occurred through electrostatic interactions between the positively charged adsorbate and the negatively charged surface of the cerium-modified adsorbent. The kinetic data obtained for the removal experiment revealed that the removal was fast, and the maximum adsorption was achieved within 2 h. Moreover, the immobilization of fipronil was strongly pH-dependent. Adsorptive removal of fipronil increased with the raise of pH from 2 to 3. The hydrogen ions which are positively charged enhanced the electrostatic interactions between the anionic oxygen of the adsorbent and the positively charged pesticide. The later decrease of adsorption efficiency in the pH range of 4–10 was explained by the deprotonation of the adsorbate which suppresses the electrostatic force between the pesticide and the adsorbent surface [122].

De Smedt and coworkers [120] investigated the adsorption of mobile (bentazone and clopyralid) and immobile pesticides (imidacloprid, and metalaxyl-m) onto different adsorbents. They reported that the removal of clopyralid and bentazone by all zeolites was rather low, probably due to the high water solubility and low octanol–water partition coefficient. On the other hand, for imidacloprid and metalaxyl-m, almost 100% removal of immobile pesticides from water solutions was possible to achieve (Table S5). Moreover, zeolite beta and zeolite Y showed the highest adsorption capacities, related to other zeolites, which may be clarified by their hydrophobicity, large surface area (high porosity), and pore-limiting diameter. Kinetic studies revealed that adsorption was fast in the case of both beta and Y zeolites, but beta zeolites were capable for removing the pesticides faster. The pesticide’s mobility also had influenced the adsorption rate—nonionic pesticide (metalaxyl-m) showed a stronger affinity for the zeolites [120].

The removal of carbendazim and imidacloprid by 5 different zeolites was studied by Chen et al. [104]. The FeNaY zeolite was obtained by impregnating NaY zeolite with 3.5% of Fe, FeY zeolite by ion exchange, and the NTY sample was dealuminated. The results showed that compared to NaY zeolite, reduction in hydrophilicity and increase in the Si/Al ratio, which was a result of dealumination process (NTY sample), caused significantly higher adsorption capacity toward carbendazim. MCM-22 revealed excellent adsorption capacity toward carbendazim (100%), which may be attributed to its structural characteristics—it has an Si/Al ratio of 10 that suppresses the competing adsorption of water molecules and big cavities. Its pores in the shape of gourd enable the pesticide to fit in and prevent its movement; this enables MCM-22 to successfully entrap nitrosamines [144]. However, on the other hand, imidacloprid cannot be effectively immobilized by none of the proposed adsorbents [104].

##### Triazine and Urea Pesticides

Zeolites Y are very popular among pesticide adsorbents due to their structure and chemical properties. Therefore, commercial zeolite Y in its hydrogen form (zeolite HY) has been investigated in a few studies for the removal of simazine pesticide [123,124,125] (Figure 7). The results indicated that the amount of simazine adsorbed onto zeolite HY varied depending on the pH of the solution. At acidic pH, the adsorption was negligible but increased rapidly at a pH up to 6.5 and then significantly declined at pH 7.0. In water solution, zeolite retained only Brønsted acid centers, while simazine behaved as a base due to the presence of the lone pairs of electrons on the N atoms. Thus, the authors believed that at specific conditions—when acid sites of HY zeolite may be neutralized by the hydroxyl anions, the Si (O−) Al groups are developed, with OH− being a stronger base than simazine, and hydronium cations develop substituted ammonium species by interaction with the nitrogen lone pair electrons of simazine—immobilization of simazine involves a typical acid–base reaction. Kinetic experiments revealed that the removal of simazine was initially fast, and then steadily slowed down and attained an equilibrium after about 24 h. Moreover, the analysis of the influence of the initial pesticide concentration allowed assuming that due to its steric hindrance the simazine molecule requires itself to be properly oriented in order to have access through the voids of the HY zeolite structure. At relatively low simazine concentration, and with weak pesticide-zeolite interactions, the amount of properly oriented pesticide molecules is low, which justifies the low efficiency of adsorption. At higher simazine concentration, more simazine molecules show proper orientation, and thus, the amount of simazine adsorbed onto HY zeolite is decidedly higher [123,124,125].

The changes in Brønsted sites induced by the alkaline treatment of zeolite samples also significantly influenced the adsorption of mesosulfuron-methyl on the hierarchical HMOR and HZSM-5 zeolites prepared by post-synthesis desilication by alkaline treatment followed or not by acid leaching of the microporous parent zeolite [108]. The alkaline treatment was performed under stirring at temperatures of 60 and 85 °C for HZSM-5 (HZSM-5-60 and HZSM-5-85, respectively) and at 70 and 90 °C for HMOR (HMOR-70 and HMOR-90), while acid treatment was performed to eliminate the extra-framework aluminum and/or silica species (samples with additional suffix “Ac”). The authors indicated that the adsorption of mesosulfuron-methyl is linked to the accessibility of the acid sites. At pH 6, the internal porosity of zeolite has a negative charge as the Brønsted acid sites release their protons in the solution, and thus, the acid-neutral form of mesosulfuron-methyl can interact with the zeolite. The best adsorption properties were found for the desilicated and acid-treated samples which is caused by effortless access to a greater number of Brønsted acid sites. Nevertheless, the adsorption of mesosulfuron-methyl is not only determined by the amount of Brønsted acid sites but also linked to the mesoporous volume and zeolite type. Its adsorption is significantly higher in hierarchical zeolites, due to the creation of interconnected mesopores in the microporous network during the desilication treatments [108].

Bottero et al. [126] adsorbed atrazine, another popular herbicide, onto zeolite Y and ZSM-5 in their study. The results clearly stated that the adsorption efficiency of zeolite ZSM-5 was meaningfully greater than zeolite Y; however, both adsorbents exhibited poor affinity toward atrazine, compared to, for example, activated carbon, the adsorption capacity of which is at least 100 times larger. The authors compared these results with the adsorption of atrazine from natural water and proved that the retention of apolar adsorbates, such as atrazine, could relate to the quantity and quality of the adsorbed background organics, which in the adsorbed form may play the role of “hosts” for pesticides [126].

The results of the experiments conducted on atrazine removal onto zeolite Y by Sirival [127] confirmed the work of Bottero et al. [126]. Sirival [127] used three different types of zeolites with various Si/Al ratios—3.61 (Y), 8.61 (Y-10), and 111.35 (Y-100)—and HDTMA-modified versions of each of them for the removal of atrazine and linuron. The author found that linuron showed higher adsorption capacity than atrazine, probably because of its smaller particles. Zeolites used in this study had a pore size of 7.4 Å, which was accessible for linuron (thickness 6.12 Å) but not for atrazine (thickness 9.6 Å). Moreover, a higher Si/Al ratio significantly enhanced the adsorption of linuron and atrazine, which is related to the dealumination potential, low polarity, and high hydrophobicity. Modification of zeolite Y with HDTMA surfactant decreased the adsorption efficiency toward linuron and atrazine, which may be due to the steric effect and pore blocking by the surfactant. However, for zeolites Y-10 and Y-100, the results observed for the adsorption of atrazine and linuron onto MY-10 and MY-100 were similar or slightly better, due to the increased hydrophobic force in the modified zeolites (Table S6) [127].

Compared to the zeolite Y (studies mentioned above), zeolites A and X, synthesized from Egyptian kaolin appear to have higher adsorption capacity regarding atrazine [128]. The results implied that the adsorption of atrazine was much faster in zeolite X—equilibrium was attained after 60 min, while for zeolite A it took more than 6 h. Moreover, at lower atrazine concentrations, zeolite X exhibited much better sorption than zeolite A. It may be connected to the greater content of positive cations in zeolite X. Cation acts as strong localized positive charge and attract atrazine. The surface area of zeolite X is 688 m^2^/g and that of zeolite A is 333 m^2^/g; however, most of the surface area in zeolite X was inaccessible to atrazine, due to the size of the pores. Thus, at a great initial concentration of atrazine, the adsorption efficiency was lower. On the other hand, zeolite A has a lower surface area but a higher content of wide pores, which were accessible to atrazine molecules [128].

Nicosulfuron is a general use pesticide that is relatively non-toxic; nevertheless, due to its widespread use, immobilization of nicosulfuron is a subject of concern of scientists [129]. In a study, zeolite BEA and silver tungstophosphate composites were developed using three different procedures: two-step impregnation (BAgPW-TI), ion exchange (BAgPW-IE), and physical mixing (BAgPWPM). In addition, composites of BEA zeolite and AgPW were prepared using zeolite and AgPW in three different mass ratios (2/1, 4/1, and 10/1). The authors of the study investigated the effect of the adsorbent amount on the immobilization of nicosulfuron. In a small volume of suspension (2 mL of solution per 20 mg of the sample), the pesticide was readily adsorbed onto the investigated samples and the amount of adsorbed pesticide was high, which was predictable taking into account the specific surface area of the studied adsorbents. At a higher suspension volume (10 mL of solution per 20 mg of the adsorbent), differences were evident in the amount of adsorbed pesticide for materials synthesized by different procedures. The samples prepared by physical mixing exhibited the lowest adsorption capacity, even lower than that of pure zeolite, while those obtained by the two-step impregnation exhibited a higher rate of pesticide adsorption with raising AgPW content. It is believed that the possible mechanism of adsorption involves H bonding, with the misleading effect of Keggin anion caused by the reactions between its own oxygen atoms and the hydrogen or silver atoms on the adsorbent surface. Moreover, in the case of composite BAgPW-TI2, which had the greatest salt amount, it was evident that there were existed agglomerate anions that did not contact directly with the adsorbent surface. The two-step impregnation enabled the development of AgPW salt as the predominant form at the adsorbent surface, which significantly increased adsorption compared to the ion-exchange process that left some of the Keggin in the form of HPW [129].

Similar studies regarding removal of nicosulfuron by tungstophosphoric acid and zeolite BEA composites, which were prepared by wetness impregnation, followed by ultrasonication and calcination, were conducted by Bajuk-Bogdanović et al. [130]. All the analyzed samples showed satisfying adsorption capacities of nicosulfuron; however, the sonicated samples exhibited better adsorption capacity compared to the as-synthesized and calcined ones. It was also implied that the content of HPW (tungstophosphoric acid) in the composite and the surface charge did not influence the removal behavior; rather, the homogeneity of the surface coverage with the HPW and the distribution of reactive sites. The possible mechanism of immobilization could be H bonding between nitrogen in the secondary amine of nicosulfuron and H in the zeolite extraframework locations. Moreover, the greater adsorption efficiency of the HPW composites compared to the zeolite BEA could be justified by extra interactions occurring between the HPW anions and the nicosulfuron species (Table S6) [130].

Nicosulfuron may also be also effectively removed by polyaniline/BEA zeolite composites [131]. Composites of BEA zeolite and polyaniline (PANI) were prepared by using aniline and BEA zeolite in two different mass ratios (1/1 and 1/5), and the obtained samples were labeled as PANI/BEA 1/1 H_2_O and PANI/BEA 1/5 H_2_O, respectively. Additionally, the composite materials PANI/BEA 1/1 H_2_SO_4_ and PANI/BEA 1/5 H_2_SO_4_ were synthesized with the same mass ratios by oxidative chemical polymerization of aniline in a sulfuric acid solution. Subsequently, each sample was deprotonated (samples denoted with “d”). The results indicated that the adsorption capacities of all the samples vary between 5.5–25.4 mg of pesticide per gram of the chosen adsorbents. The highest removal was found for the protonated sample prepared in water, with aniline and zeolite in the mass ratio of 1/1 (PANI/BEA 1/1 H_2_O). Generally, all the protonated forms of zeolite composites were more effective in the removal of pesticide than the deprotonated ones. The lowest adsorption efficiency was observed for the sample PANI/BEA 1/5 H_2_O d., which was even lower than that of the pure BEA zeolite (17 mg/g) (Table S6). Furthermore, the protonated composites synthesized in acid solution showed greater homogeneity of the active sites in comparison with the samples synthesized without acid, and thus exhibited a slightly higher adsorption capacity for nicosulfuron. The authors suggested that the possible mechanism of immobilization at pH 5 was H bonding between pesticide and the bridging hydroxyls of BEA and the nitrogen-containing groups of PANI (for both protonated and deprotonated samples), and also the electrostatic interactions between the anionic form of nicosulfuron and the positively charged PANI emeraldine salt chains (in the case of the protonated forms) [131].

For the adsorption of nicosulfuron onto ZSM zeolite and KPW (potassium salts of 12-tungstophosphoric acid) composites, the method of synthesis appears to have a significant influence. Jevremovic and coworkers [132] prepared two synthesis methods that differ the in order in which the respective salts are added: the K/PW procedure and PW/K procedure. In the first method zeolites with various SiO_2_/Al_2_O_3_ ratios (30, 50, or 80) were treated with K_2_CO_3_ in the first step and HPW in the second step. Samples are denoted as Z(30, 50, or 80)K/PW-D for dried ones and Z(30, 50, or 80)K/PW-C for calcined composites. In the second procedure, zeolites were firstly mixed with HPW, and then with K_2_CO_3_. Samples are labeled as Z (30, 50, or 80)PW/K-D for dried ones and Z(30, 50, or 80)PW/K-C for calcined. The results indicate that unmodified zeolites have the lowest affinity to the nicosulfuron pesticide, and it can be concluded that the active KPW phase has a key influence on the effectiveness of the removal of pesticides. The greater removal of modified samples compared to unmodified adsorbents may be justified by the extra reactions between PW anions and nicosulfuron particles. The reason for the predominancy of the method in which potassium cation is added firstly and PW secondly is the fact that potassium cation is evenly distributed over adsorbent cation-exchange locations, and when PW is added, a homogeneous layer of KPW may be attained. The second method (PW/K) is influenced by electrostatic repulsing of Keggin anion and adsorbent surface, and KPW salt development and distribution are not as effective as in the first procedure. Moreover, the Si/Al ratio also has a significant impact on the adsorption properties of ZSM zeolites. Greater aluminum content and charge of the surface result in more effective potassium cation exchange and posterior KPW development than in samples with higher Si/Al ratios [132].

Adsorption of isoproturon—mobile, non-ionic pesticide—onto 7 different types of zeolites was examined by De Smedt et al. [120]. Results indicate that isoproturon was absorbed very quickly. For zeolites BEA and Y, it took 15 and 30 min, respectively, to achieve an equilibrium state. Moreover, due to being non-ionic, isoproturon has a stronger affinity for the zeolites, than ionic pesticides (compare Tables S5 and S6). Generally, nonionic pesticides are rather immobile compared to ionic ones, and thus their affinity for zeolites is greater [145], which explains a higher adsorption intensity, compared with the other pesticides. In addition to that, a higher adsorption rate observed for zeolites BEA and Y can be explained by their hydrophobicity, high porosity, and pore-limiting diameter [120].

##### Glyphosate-Based Pesticides

Glyphosate is one of the most widely used herbicides applied to control broadleaf weeds and grasses and thus is a subject of many adsorption experiments. Zavareh et al. [112] studied the adsorption of glyphosate onto zeolite 4A and Cu^2+^ ion-exchanged zeolite with (Cu-modified zeolite 4A). The modified zeolite showed a greater adsorption efficiency than pristine zeolite at all pH values, particularly at neutral ones (6–8). However, both adsorbents exhibited better adsorption properties in acidic conditions. In the case of pure zeolite 4A, this can be explained by electrostatic interactions. The pH at the point of zero charge (pH_pzc_) was established to be 3.9, so at a lower pH the surface of the adsorbent was positively charged. Glyphosate is deprotonated at pH 2.3 and occurs in the form with two negative charges and one positive charge (one negative net charge). The electrostatic interaction between the anionic form and the adsorbent’s surface may be responsible for the considerable adsorption of glyphosate at pH 3. For Cu-zeolite 4A, the adsorption efficiency slightly increased at pH 3 but decreased steadily with the raise of pH up to 6 and remained practically unchanged at pH 6–9. Its pH_pzc_ was established to be 6.2. The authors suggested that the behavior of Cu-4A can be associated with chemical and electrostatic reactions. At pH < 6.2, those mechanisms occurred in the same direction which enlarged the efficiency of adsorption of the modified zeolite. At pH 6.2–9, the chemisorption of glyphosate via complex formation with Cu^2+^ in the adsorbent structure allowed constant adsorption, while at pH >9, the formation of copper hydrate could be responsible for the decrease of glyphosate adsorption [112].

Impregnation of zeolites with single ions is a popular way to modify their properties; however, salts may also be used for this purpose. Potassium tungstophosphate supported on BEA zeolite was synthesized in situ and used for glyphosate adsorption in a study [110]. The zeolite was modified with 10, 20, and 30 wt.% of KPW (potassium salts of 12-tungstophosphoric acid), and some samples were further calcinated. The results of the experiments conducted with the prepared samples indicated that the removal of glyphosate was significantly affected by the synthesis procedure—calcinated adsorbents showed higher adsorption capacities. Sample BKPW-2C exhibited the highest adsorption capacity for glyphosate, probably due to the reactions between KPW and pesticides via H bonds developed between the secondary amine in the protonated form of glyphosate and the unbonded oxygen atoms in the KPW structure. Moreover, it was implied that glyphosate was adsorbed mainly as a zwitter-ion rather than as a monoanionic form at the adsorbent surface containing KPW, which affected the adsorption behavior of the investigated composite materials, rather than acidity; thus, the higher the KPW content, the higher the adsorption capacity [110].

Similarly, to the KPW-modified zeolite, for polyaniline-ZSM5 composites the highest polyaniline content is also related with the highest adsorption capacity toward glyphosate. Polyaniline-ZSM5 composites were prepared by the oxidative polymerization of aniline in water and an aqueous solution of sulfuric acid [111]. The synthesized samples were labeled as PZ1/1, PZ1/5, and PZ1/10 with respect to the ZSM-5/aniline weight ratios of 1, 5, and 10, respectively. The samples prepared in the presence of strong acid, H_2_SO_4_, were denoted with an additional suffix “S” in their label, while the deprotonated forms were indicated with “d”. The results showed that PANI/ZSM-5 materials, in both protonated and deprotonated forms, exhibited great adsorption capacities for glyphosate (Figure 8). Among the obtained materials, the poorest removal of glyphosate was found for the materials with the greatest content (86%) of ZSM-5; thus, the highest the PANI content, the highest the adsorption capacity. Moreover, deprotonation appeared to enhance the adsorption of glyphosate. The authors suggested that in the case of the materials with the lowest content of zeolite, the adsorption was influenced mainly by the molecular structure of polymer chains, level of protonation, oxidation state, chain branching, the number of structure defects, and interactions occurring between PANI and glyphosate such as hydrogen bonding or acid–base interactions [111].

## 4. Regeneration and Recyclability

### 4.1. Mesoporous Silica Materials

In order to evaluate the viability and practical capability of adsorbents used for pesticide immobilization, it is essential to examine the adsorption effectiveness after successive adsorption–desorption of the pesticide. Results gained by Otalvaro et al. [105] indicate that the adsorption efficiency of 0.24APTES-MCM-41 strongly decreased in the first cycle and after the third cycle reached 25% of original adsorption. Nevertheless, it is worth to mention that even after 2 cycles of reuse, the adsorption efficiency was still greater than for the pristine MCM-41. The stability of adsorbent seems to have a key impact on the reusability, probably due to the partial hydrolysis of the APTES in water. In addition, the lack of FTIR bands of adsorbed 2,4-D indicates that almost all pesticide was removed during adsorption–desorption processes [105].

Brigante and Avena [116] examined the influence of double-distilled water and the HCl solution onto the regeneration potential of MCM-41. Material washed with 0.2 M HCl solution preserved more than 75% of its removal efficiency after four cycles of regeneration; however, when water was used for washing, the removal efficiency was around 30%. It suggests that the protonation of surface groups on SiO_2_, prompted by washing in acid, increases the desorption of the paraquat. What is more, MCM-41 is characterized by narrow mesopore channels, which can hinder the transport of the positively charged paraquat ion, so that the pesticide molecules may be clogged in the pores and voids, and their release may be constricted [116].

The major factor that influenced recyclability plays the polarity of the solvent used for regeneration, due to its influence on the solubility, and H-bonding interactions [119]. Desorption of bentazone from MCM-41 was prepared with aqueous solutions (H_2_O, pH 7) and organic solvents (CH_3_OH, CH_3_CN, ethanol, 2-propanol) or mixtures (20 mM NaOH, 80 % CH_3_OH). Results indicate that the most efficient for all the organic solvents tested is ethanol, which exhibits the greatest recovery percentages. Although the authors did not state the clear correlation between polarity and the recovery of pesticide, CH_3_CN and 2-propanol, which exhibits the lowest polarity values, showed the poorest desorption. The solvent may interact with adsorbed pesticide or surface of the adsorbent and thus influence the H bonding. Methanol exhibits the highest desorption efficiency among all protic solvents (CH_3_OH, ethanol, and 2-propanol). The highest recovery of all tested adsorbents was observed for the mixture of NaOH and CH3OH which indicates that desorption of bentazone on the way of dissociation may be the most effective approach [119].

Rivoira and coworkers [109] used NaOH for the regeneration of Fe-NH2-SBA-15 with absorbed glyphosate. The results obtained during 5 cycles of adsorption and desorption of pesticide clearly indicate that adsorbent can be effectively regenerated and reused at least five times. The authors of the work suggest that it is the first time when complete regeneration with unaltered performances was achieved for this kind of adsorbent [109].

A different way of adsorbent regeneration was proposed by Tian et al. [100], who used the thermal decomposition procedure for the regeneration of the tested sorbents, in order to completely decompose pesticides without producing toxic byproducts. It was indicated that at a comparatively low temperature (450 °C) and quite a short time (2 h), almost 100% DDT can be degraded. The decomposition efficiency of DDT, DDE, and DDD increased with increasing reaction temperature, and at 350 °C, DDT was completely converted. Thus, authors suggested that the organic particles immobilized on silica adsorbents can be easily destroyed by heating. Regenerated adsorbents were analyzed by nitrogen adsorption–desorption. SBA-15 was most stable and retained its surface area and porosity. HMS retained most of its pores after treatment. But oppositely, the porosity of MCM-41 and MCM-48 were significantly decreased due to their low hydrothermal stability [100].

### 4.2. Synthetic Zeolites

Vasiljevic et al. [110] performed experiments concerning the possibility of regeneration of BKPW adsorbents, since BKPW composites were demonstrated to be stable at 300 °C and glyphosate degrade at over 198 °C. FTIR investigation of samples before and after thermal treatment confirmed the pesticide removal. Characteristic bands attributed to glyphosate vibrations were missing in the spectra of samples after thermal treatment, which is evidence of pesticide removal. The second round of adsorption indicated that blockage of adsorption centers during thermal degradation resulted in lowering the adsorption capacities by approximately 66% compared to maximum adsorption results in the first round of adsorption [110].

On the other hand, Zavareh et al. [112] conducted experiments of chemical regeneration of Cu-4A zeolite, with previously absorbed glyphosate. The samples of adsorbent were treated with CuSO4 solution with five cycles of adsorption and desorption. Results indicate that only a minor decline (7%) in adsorption efficiency was noticed after 5 cycles—most of the glyphosate species were desorbed with the exchanged Cu^2+^ ions. Thus, a high regeneration capability was gained for the tested materials [112].

## 5. Discussion

Extensive research of the adsorption processes has conducted to the development of many empirical isotherm equations, which are useful to find theoretical maximum adsorption capacity and to designate the adsorption process as monolayer or multilayer. There are few isotherms that have been commonly used—the Langmuir, Freundlich, Langmuir–Freundlich, and Halsey. The original form of isotherms is non-linear but, usually, some transformations are performed to simplify them. However, it must be here highlighted that this conversion may be misleading since it unavoidably changes the distribution of the error function. Thus, it is suggested to use non-linear version of isotherms to avoid such problems [22,146,147,148,149].

The Langmuir equation [102]:

(2) $$q_{e}=\frac{Q_{0}K_{L}C_{e}}{1+K_{L}C_{e}}$$

q_e_ is the pesticide adsorption capacity at equilibrium (mg/g); C_e_ is the equilibrium concentration of pesticide (mg/L); K_L_ is Langmuir constant (L/g), and Q_0_ is the maximum adsorption capacity (mg/g).

The Freundlich equation [102]:

(3) $$q_{e}=K_{F}C_{e}^{1/n}$$

q_e_ is the pesticide adsorption capacity at equilibrium (mg/g); K_F_ is Freundlich constant that refer to the adsorption capacity at unit concentration; C_e_ is the equilibrium concentration of pesticide (mg/L), and 1/n is adsorption intensity of the solute on the adsorbent.

The Langmuir–Freundlich equation [150]:

(4) $$q_{e}=\frac{Q_{0}{\left(K_{a}C_{e}\right)}^{n}}{{\left(K_{a}C_{e}\right)}^{n}+1}$$

q_e_ is the pesticide adsorption capacity at equilibrium (mg/g); Q_0_ is the maximum adsorption capacity (mg/g); C_e_ is the equilibrium concentration of pesticide (mg/L); K_a_ is the affinity constant for adsorption (L/mg), and n is the index of heterogeneity.

The Halsey isotherm [52]:

(5) $$q_{e}=\exp\left(\frac{\ln K_{H}+\ln C}{n}\right)$$

q_e_ is the pesticide adsorption capacity at equilibrium (mg/g); C_e_ is the equilibrium concentration of pesticide (mg/L), and K_H_ and n are the Halsey isotherm constant and exponent, respectively. 

The results obtained by various researchers indicate that for most silica-based adsorbents adsorption data fits Langmuir or Freundlich models (more than 85% of all of the studies reporting isotherm analysis). Langmuir’s and Freundlich’s isotherms are one of the most frequently used for the description of adsorption of organic pollutants onto porous materials. Langmuir isotherm describes the monolayer adsorption that occurs onto a surface with a designated number of equal adsorption sites that are equal in affinity toward species adsorbed molecules and equal adsorption energy. The Langmuir equation can be used for homogeneous surfaces. The Freundlich adsorption isotherm is an empirical equation that can be used to define the adsorption properties for a heterogeneous surface. Thus, the difference between the two isotherms is the assumption of chemisorption for a monolayer with Langmuir’s model, whereas the Freundlich isotherm is empirical and does not rest on any strict assumption on the nature of the adsorption. The Langmuir model applies to chemisorption, unlike Freundlich adsorption that does not differentiate between chemisorption and physisorption involving weak Van der Waals forces. 

The Langmuir model fits better the description of adsorption of paraquat [114,115,116], 2,4-D [105], and diazinon [102]. On the other hand, some equilibrium data fits better the Freundlich model like adsorption of avermectin [113], bentazone [119], pentachlorophenol [51,97], and mesosulfuron-methyl [107]. Some experiments revealed that the adsorption isotherm model that fits the adsorption strongly depends on the modification of the adsorbent surface. Adsorption of DEET onto silylated MCM-41 samples can be described by the Langmuir model while for the as-synthesized MCM-41 and HMS solids Fowler–Guggenheim model was better [118]. Similarly, adsorption of 2,4-D was described as monolayer adsorption; however, adsorption onto SBA-15/80 corresponded better to the Langmuir model, while adsorption onto SBA-15/100 was described better by the Halsey model [52].

Among the adsorption isotherms that can be used for the description of adsorption of pesticides onto unmodified and modified zeolites, Langmuir’s and Freundlich’s models also dominate (around 90% of reported data). Langmuir model was applicable for adsorption of paraquat [106,114,115,117], α-endosulfan [121], 2,4-D [106], and fipronil [122]; Freundlich model may be used for explanation of adsorption of simazine [123,124], nicosulfuron [130], and mesosulfuron-methyl [108], while Langmuir–Freundlich model was applicable for adsorption of nicosulfuron [131]. Adsorption of glyphosate may be attributed to the different adsorption isotherm models depending on the adsorbent used. For BEA zeolite and potassium tungstophosphate-modified BEA zeolite, Langmuir model fits better [110]; for 4A and Cu-4A [112], Freundlich isotherm shows better results, while for HZSM-5, polyaniline-ZSM5 composites, both Freundlich and Langmuir–Freundlich models appear to have the best fit [111]. However, it is necessary to state that although some articles consider a wide range of different types of isotherms, most of them were focused only on Langmuir and Freundlich models; thus, the usage of, e.g., the Langmuir–Freundlich model should be deliberated as in articles of Milojevic-Rakic et al. [111] and Jevremović et al. [131].

The results obtained by various researchers indicate that the type of pesticide may have an essential influence on the shape of the adsorption isotherm. It is more clearly conspicuous for mesoporous silica materials than for zeolites. Although the surface properties of, e.g., unmodified MCM-41 are the same for all pesticide adsorption experiments, the adsorption of paraquat fits the Langmuir model [114,115,116] while adsorption of pentachlorophenol fits the Freundlich model [51,97]. Thus, it may be concluded that properties of the surface of adsorbents and their modifications may have a lesser role than the properties of adsorbates. Moreover, the synthesis methods and preparation of the samples appear to be important. Generally, the more homogeneous the surface, the more it fits the Langmuir model. According to Giles classification [151], all the presented isotherms exhibit an S-Shape [52,97,107,108,124]. These isotherms were described by the Freundlich isotherm model. S-Shape isotherm is usually attributed to the adsorption of polar molecules from an aqueous solution, where non-homogeneous surface adsorption sites force the adsorbate–adsorbent interactions. If a certain amount of the adsorbate molecules is already attached to the surface of the adsorbent, the additional molecules can be adsorbed on the surface more easily. This phenomenon suggests the occurrence of side-by-side intermolecular attraction between adsorbed particles and implies that a stable layer of adsorbate is formed. This may occur especially when the pesticide possesses a largish hydrophobic part and demonstrates a moderate intermolecular attraction, which may lead to perpendicular packing of the adsorbate in relation to the adsorbent surface and competition for adsorptive sites between adsorbate and solvent molecules [52,97].

The kinetics of adsorption, which indicates the time needed for the adsorbate to adsorb, is one of the most essential tools to describe the adsorption processes and mechanisms. The adsorption kinetics may be described using three theoretical models: pseudo-first-order model, pseudo-second-order model, and intraparticle diffusion model. However, it should be stated that although the pseudo-second-order rate equation, popularized by Ho and Mckay [152], is probably the most popular model used to describe adsorption kinetics, utilization of this model is controversial [149,153,154]. Zhang in his communication in ChemComm journal from 2011 [153] brought attention to the improper formulation of data analyses which can lead to an inflation of the meaning of statistical correlations and result in making misleading conclusions. The common misapplication of a second-order kinetic model in the literature exposes that many scientists are not aware of the dangers of misguided correlation.

The pseudo-first-order model [112]:

(6) $$q_{t}=q_{e}[1-\exp(-k_{1}t)]$$

q_t_ is the amount of adsorbed pesticide (mg/g) at time t (min); q_e_ is the pesticide adsorption capacity at equilibrium (mg/g), and k_1_ is rate constants for pseudo-first-order model.

The pseudo-second-order model [112]:

(7) $$q_{t}=\frac{q_{e}^{2}k_{2}t}{1+q_{e}k_{2}t}$$

q_t_ is the amount of adsorbed pesticide (mg/g) at time t (min); q_e_ is the pesticide adsorption capacity at equilibrium (mg/g), and k_2_ is rate constants for pseudo-second-order model.

The intraparticle diffusion model [113,116]:

(8) $$q_{t}=k_{3}t^{0.5}+C$$

q_t_ is the amount of adsorbed pesticide (mg/g) at time t (min); q_e_ is the pesticide adsorption capacity at equilibrium (mg/g); C is the intercept, and k_3_ is rate constants for intraparticle diffusion model.

Among all the articles cited in this work, where authors conducted kinetic modelling, more than 90% of the studies reporting a kinetic analysis reported a best fit to the pseudo-second order than to pseudo-first-order model [51,52,100,102,106,107,108,112,116,120,122,123]. According to the literature, assigning adsorption data to the pseudo-second-order model implies that in adsorption of pesticides onto zeolites and mesoporous silica materials, chemisorption may predominate. The pseudo-second-order model is built on the presumption the chemical adsorption or chemisorption may be the factor that limits the rate of adsorption, while the pseudo-first-order model assumes that one ion is adsorbed onto one unoccupied adsorption site on the adsorbent’s surface and the reaction is more inclined towards physisorption [51,52,106,107,113,152,155]. However, it is risky to draw those conclusions since the problem has been discussed by several researchers [156,157,158], who assert that prediction of adsorption mechanisms cannot be drawn based on the applying of the kinetic models (i.e., the pseudo-first-order and pseudo-second-order models). Adsorption mechanisms can only be recognized by using analytical techniques like FTIR, SEM, nitrogen adsorption–desorption isotherms, Raman spectroscopy, TGA/DTA, DSC, or by proper and careful predicting of the chemical or physical nature and interactions between the adsorbate and the active sites on the surface of the adsorbent [149,157,158,159]. 

Intra-particle diffusion kinetics models have also been applied for the description of some pesticides’ adsorption data [51,113,116]. In contrast to the pseudo-first-order and pseudo-second-order models, the intra-particle diffusion model is suitable for identifying the processes and adsorption mechanisms and predicting the rate-controlling step [149]. The diffusion model is based on one or more of the following mechanistic steps: external mass transport across the boundary layer surrounding the particle; diffusional mass transfer within the internal structure of the adsorbent particle by a pore, surface, branched pore, or a combination of these mechanisms; adsorption at a surface site [154]. 

**Figure 9:**
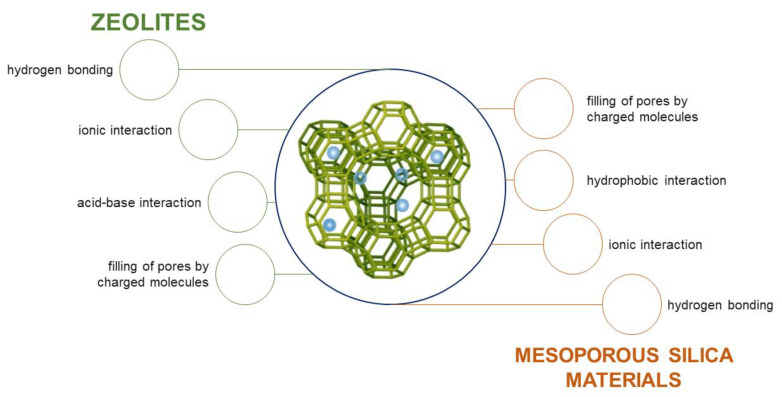
Mechanisms of pesticides immobilization onto modified and unmodified zeolites and mesoporous silica materials.

Literature data indicate that the mechanisms of pesticide adsorption onto mesoporous silica and zeolites are similar (Figure 9). Most pesticides are immobilized onto mesoporous silica materials by weak interactions with the external wall surfaces and the filling of mesopores by charged pesticide molecules [51,52,97,98,100,102,105,107,109,116,119]. It is assumed, that those interactions involve electrostatic attractions and outer-sphere–complexes (or ionic pairs) formation between the pesticide’s functional groups and the active sites on the surface of silica. They are strongly influenced by the pH of the pesticide solution. Different pesticides have different charges, so repulsion between the pesticide molecule and the surface of the adsorbent may inhibit adsorption. One example is the removal of glyphosate by SBA-15 which is extremely high at low pH conditions; however, with rising pH value, the effectiveness of adsorption decreases [109]. Similarly for adsorption of 2,4-D onto unmodified and modified SBA-15. Ineffective immobilization of 2,4-D at higher pH values is provoked by the dominant electrostatic repulsions between pesticide and adsorbents surfaces [52]. Moreover, the density of OH groups on the surface of mesoporous silica depends on the type of materials (e.g., for SBA-15 and MCM- 48 the OH density is usually high) [100,160]. OH density increases the speed of adsorption, since the hydrophilic molecules may be adsorbed on the way of hydrogen bonding onto the inner pore surface; thus, the higher density of the OH groups, the faster the adsorption process, which is in agreement with the kinetic studies. Adsorption of most pesticides is a very rapid process—for more than 90% of studies reporting a kinetic study, pesticides were adsorbed in the first few minutes, and after approximately 2 h adsorption is usually completed [51,52,97,100,105,107,116,119]. Thus, hydrogen bonding, electrostatic interactions, and hydrophobic interactions can be recognized as the main mechanism for the adsorption of pesticides onto silica materials.

In the case of synthetic zeolites hydrophobicity, and Si/Al ratio were found to significantly affect the removal of pesticides. Generally, a higher Si/Al ratio enhanced the pesticide adsorption, which is related to the dealumination potential, low polarity, distribution of negative charge in the zeolite frameworks, and high hydrophobicity. Similar to the mesoporous silica, mechanisms of adsorption of pesticides onto zeolites may involve weak electrostatic interactions, the filling of pores pesticide molecules, and also typical acid–base reactions [106,108,112,120,122,123,124,131]. The zeolite in water possess only Brønsted acidic centers, since the potential Lewis acidic centers have reacted with water [161]. Thus, if pesticide possesses the electron lone pairs (e.g., simazine has electron lone pairs located on the nitrogen atoms in the lateral chains of the molecule), it behaves as a base, and it may be suggested that the immobilization mechanism involves a typical acid–base reaction [107,108,123,124,129]. These interactions occur mostly at a pH where the following two factors can be balanced: (1) at alkaline pH, hydroxyl anions neutralize the acid sites of zeolite forming Si (O−) Al groups, being OH− a base stronger than pesticide; (2) at acidic pH, hydronium cations form new species by reacting with the lone pair electrons of pesticide [123,124]. Moreover, generally speaking, non-ionic pesticides are relatively nonmobile compared to ionic ones; thus, their affinity for zeolites is higher. This explains why the non-ionic pesticides usually have a better adsorption intensity, compared with the ionic molecules [120,145].

The porosity and geometry of pores have an impact on the adsorption efficiency of mesoporous silica and zeolites; however, the capability of entrapping pesticides by the adsorbents did not strictly depend on their porosity and specific surface area [51,98,100,104,117,119,120,126,128,162]. Larger pore diameter and larger pore volume are certainly beneficial for adsorption efficiency, and higher pore connectivity resulted in a higher adsorption rate. Nevertheless, as indicated by the comparison between NaY and MCM-21 zeolites, although they possess high porosity, the adsorption efficiencies were different. A gourd-shaped pore structure in MCM-22 was more suitable for carbendazim to enter and against its release. On the other hand, although NaY zeolite has a relatively large pore size and the super cage structure, it cannot effectively entrap pesticides due to its hydrophilicity. Thus, one of the key factors for the adsorption of organic compounds in pores of adsorbents can be credited to the chemical structure of these organic molecules and their geometric fit within the shape of pores [98,104,128].

There are many adsorbents that were analyzed for pesticide removal. The most popular are active carbons, graphene-based and chitosan-based adsorbents, biochar, and clays. Zeolites and mesoporous silica materials exhibit good adsorption capacities that may be comparable to other known adsorbents. The main disadvantage of other effective adsorbents as active carbons or graphene-based adsorbents is their cost; however, it is proven that carbon-based adsorbents exhibit adsorption capacities that may be comparable to the zeolites and mesoporous silica materials [162]. The main disadvantage of mesoporous silica materials is their hydrothermal stability, which may compromise the effective long-term applicability of these adsorbents. Although typical mesoporous silica materials (e.g., MCM-41, MCM-48) exhibit excellent thermal stability, their stability in distilled water and aqueous solutions is very low compared with zeolites, e.g., A, Y, or ZSM-5 [163,164]. The structure of mesoporous silica may be easily lost in aqueous solutions during adsorption processes. However, when some techniques are used (synthesis of materials with thick pore walls, removal of silanol groups by silylation, or stabilization by a salt effect) the hydrothermal stability of the mesoporous may increase significantly and may be comparable to the stability of synthetic zeolites. [163,165,166,167].

## 6. Conclusions

This review has attempted to summarize the available information regarding the adsorption of pesticides by mesoporous silica materials and synthetic zeolites to provide the readers with an idea about the various porous silica-based adsorbents used for pesticide removal from water. Mesoporous silica materials and synthetic zeolites are effective for a broad range of pesticides. The results of the cited studies clearly indicate that the surface functionalization of adsorbents usually enhances their adsorption properties. However, because of the different conditions of experiments applied in each study and the different methods of adsorbent functionalization applied, it is hard to state with certainty which sorbent is more suitable for respective pesticide, and therefore, further research is desired to better understand the correlations between the different pesticides and adsorbents. 

Summarizing their advantages stated by various authors for the adsorption of pesticides, functionalized and unfunctionalized mesoporous silica and synthetic zeolites seem to be suitable alternatives for traditional adsorbents. The studies described in this article prove that appropriate functionalization of the adsorbent’s surface and optimization of the experimental conditions with respect to the type of pesticide may enable extremely effective removal.

## Figures and Tables

**Table 1:** Characteristics of the main groups of pesticides.

Pesticide	Mode of Action	Environmental Impact	Examples	References
Organochlorine	act as nervous system disruptors which leads to convulsions, paralysis, and death	long-term residual effect in the environment, resistant to most degradation processes	DDT (1,1,1-trichloro-2,2′bis(p-chlorophenyl)ethane), lindane, endosulfan	[9,18,22]
Organophosphorus	act as cholinesterase inhibitors causing a permanent overlay of acetylcholine neurotransmitters across a synapse which leads to paralysis and death	not persistent in the environment, susceptible to biodegradation	parathion, malathion, diazinon	[9,18,23]
Carbamates	act as cholinesterase inhibitors; mechanism of cholinesterase inhibition is species-specific and reversible	not persistent in the environment, susceptible to biodegradation	carbaryl, carbofuran, propoxur	[18,24]
Pyrethrins (natural) Pyrethroids (synthetic)	act by disrupting an insect’s nervous system which leads to a weakened state followed by death	not persistent in the environment, susceptible to biodegradation	permethrin, cypermethrin, deltamethrin	[18,25]

**Table 2:** Recapitulative table summarizing the data presented in the article.

Pesticide	Adsorbent Type	Article Section	References
Pentachlorophenol	Mesoporous silica material	3.1.1	[51,97]
DDT	Mesoporous silica material	3.1.1	[98,99,100,101]
DDE	Mesoporous silica material	3.1.1	[98,99]
DDD	Mesoporous silica material	3.1.1	[98,99]
Heptachlor	Mesoporous silica material	3.1.1	[98]
Endosulfan	Mesoporous silica material	3.1.1	[98]
Aldrin	Mesoporous silica material	3.1.1	[98]
Dieldrin	Mesoporous silica material	3.1.1	[98]
Methoxychlor	Mesoporous silica material	3.1.1	[98]
Diazinon	Mesoporous silica material	3.1.2	[102,103]
Fenitrothion	Mesoporous silica material	3.1.2	[103]
Acephate	Mesoporous silica material	3.1.2	[104]
	Zeolite	3.2.1	
Folimat	Mesoporous silica material	3.1.2	[104]
	Zeolite	3.2.1	
Phoxim	Mesoporous silica material	3.1.2	[104]
	Zeolite	3.2.1	
Chlorpyrifos	Mesoporous silica material	3.1.2	[104]
	Zeolite	3.2.1	
Dipterex	Mesoporous silica material	3.1.2	[104]
	Zeolite	3.2.1	
2,4-D	Mesoporous silica material	3.1.3	[52,105]
	Zeolite	3.2.2	[106]
Carbendazim	Mesoporous silica material	3.1.3	[104]
	Zeolite	3.2.2	
Imidacloprid	Mesoporous silica material	3.1.3	[104]
	Zeolite	3.2.2	
Mesosulfuron-methyl	Mesoporous silica material	3.1.3	[107]
	Zeolite	3.2.2.1	[108]
Glyphosate	Mesoporous silica material	3.1.3	[109]
	Zeolite	3.2.2.2	[110,111,112]
Avermectin	Mesoporous silica material	3.1.3	[113]
Paraquat	Mesoporous silica material	3.1.3	[114,115,116]
	Zeolite	3.2.2	[106,114,115,117]
DEET	Mesoporous silica material	3.1.3	[118]
Bentazone	Mesoporous silica material	3.1.3	[119]
	Zeolite	3.2.2	[120]
α-endosulfan	Zeolite	3.2.1	[121]
Fipronil	Zeolite	3.2.2	[122]
Clopyralid	Zeolite	3.2.2	[120]
Imidacloprid	Zeolite	3.2.2	[120]
Metalaxyl-m	Zeolite	3.2.2	[120]
Isoproturon	Zeolite	3.2.2.1	[120]
Simazine	Zeolite	3.2.2.1	[123,124,125]
Atrazine	Zeolite	3.2.2.1	[126,127,128]
Linuron	Zeolite	3.2.2.1	[127]
Nicosulfuron	Zeolite	3.2.2.1	[129,130,131,132]

## Supplementary Materials

The following are available online at https://www.mdpi.com/article/10.3390/ma14133532/s1. Figure S1: Structures of mesoporous silica materials. Figure S2: Framework types of selected zeolites. Zeolites X and Y mentioned in the manuscript have FAU framework type, zeolite A—LTA, zeolite ZSM5—MFI, zeolite MCM-21—MWW. Figure S3: Structures of organochlorine pesticides. Figure S4: Structures of organophosphorus pesticides. Figure S5: Structures of pesticides from other groups. Table S1: Selected adsorption capacities and other parameters for the removal of organochlorine pesticides by mesoporous silica materials. Table S2: Selected adsorption capacities and other parameters for the removal of organophosphorus pesticides by mesoporous silica materials. Table S3: Selected adsorption capacities and other parameters for the removal of different groups of pesticides by mesoporous silica materials. Table S4: Selected adsorption capacities and other parameters for the removal organochlorine and organophosphorus pesticides by synthetic zeolites. Table S5: Selected adsorption capacities and other parameters for the removal of different groups of pesticides by synthetic zeolites. Table S6: Selected adsorption capacities and other parameters for the removal of triazine and urea pesticides by synthetic zeolites. Table S7: Selected adsorption capacities and other parameters for the removal of glyphosate-based pesticides by synthetic zeolites.
