# Supplementary material for: Removal of Pesticides from Waters by Adsorption: Comparison between Synthetic Zeolites and Mesoporous Silica Materials. A Review
Source: Materials (Basel). 2021 Jun 24;14(13):3532. doi: 10.3390/ma14133532 (PMC8269501; doi:10.3390/ma14133532)
Supplement: Supplementary file 1 [file materials-14-03532-s001.zip › materials-1255974-supplementary.pdf]

materials

MDPI

Supplementary materials

Removal of Pesticides from Waters by Adsorption: Comparison between Synthetic Zeolites and Mesoporous Silica Materials. A Review

Magdalena Andrunik *, Tomasz Bajda

Faculty of Geology, Geophysics and Environmental Protection, AGH University of Science and Technology, al. Mickiewicza 30, 30-059 Krakow, Poland; bajda@agh.edu.pl
* Correspondence: magtuch@agh.edu.pl; Tel.: (48)(12) 888 45 83

Citation: Andrunik, M.; Bajda, T. Removal of Pesticides from Waters by Adsorption: Comparison between Synthetic Zeolites and Mesoporous Silica Materials. A Review. *Materials* **2021**, *14*, 3532. https://doi.org/10.3390/ma14133532

Academic Editor: José Rodríguez-Mirasol

Received: 26 May 2021
Accepted: 23 June 2021
Published: 24 June 2021

Publisher's Note: MDPI stays neutral with regard to jurisdictional claims in published maps and institutional affiliations.

Copyright: © 2021 by the author. Licensee MDPI, Basel, Switzerland. This article is an open access article distributed under the terms and conditions of the Creative Commons Attribution (CC BY) license (http://creativecommons.org/licenses/by/4.0/).

MCM-41

MCM-48

SBA-15

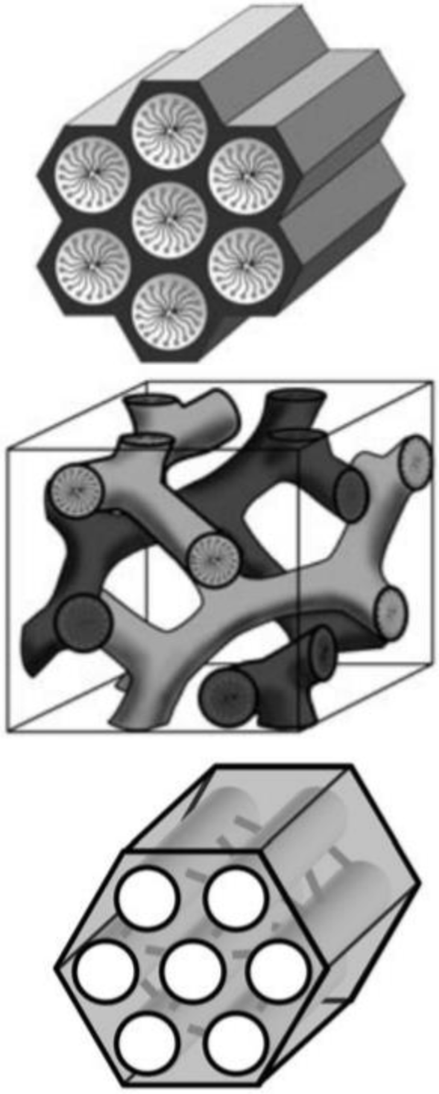

Figure S1. Structures of mesoporous silica materials.

BEA

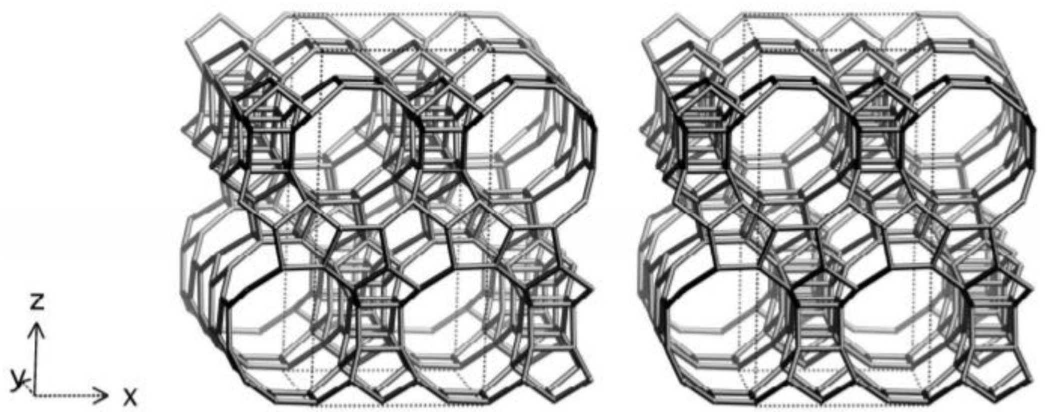

framework viewed along [010]

FAU

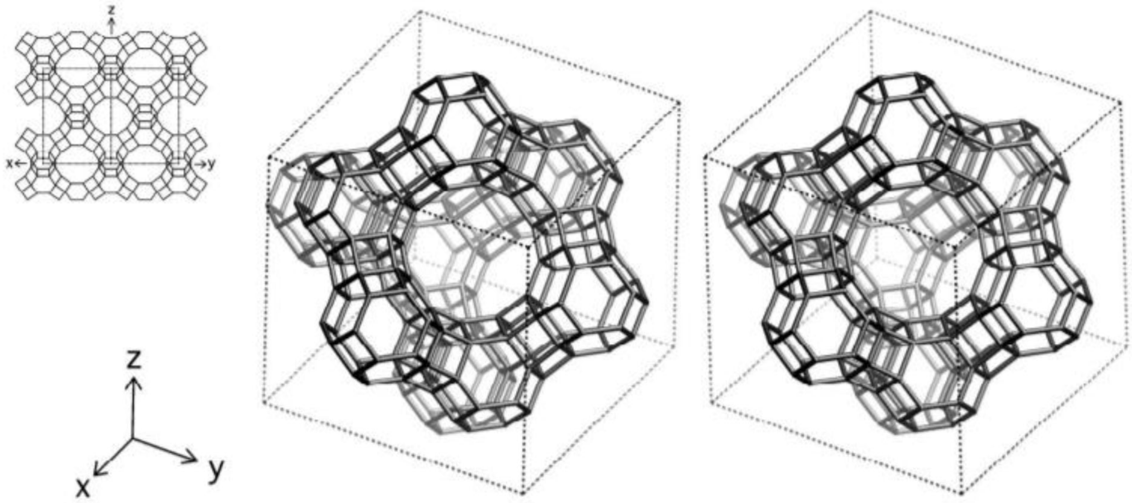

framework viewed along [111] (top left: projection down [110])

LTA

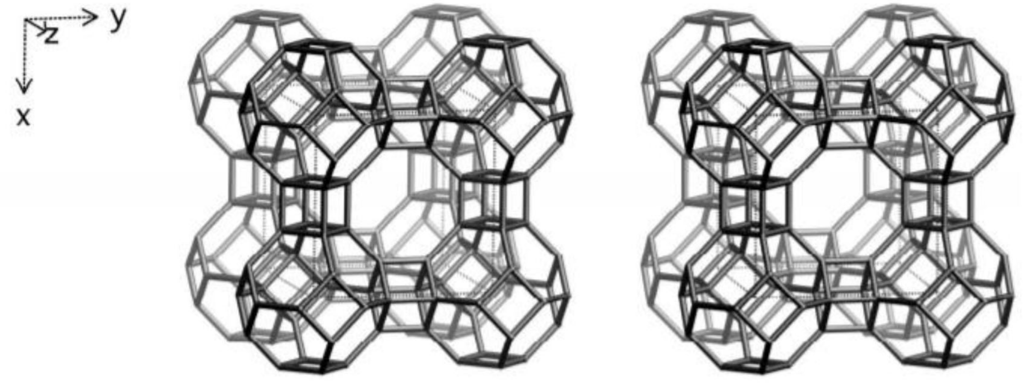

framework viewed along [001]

LTL

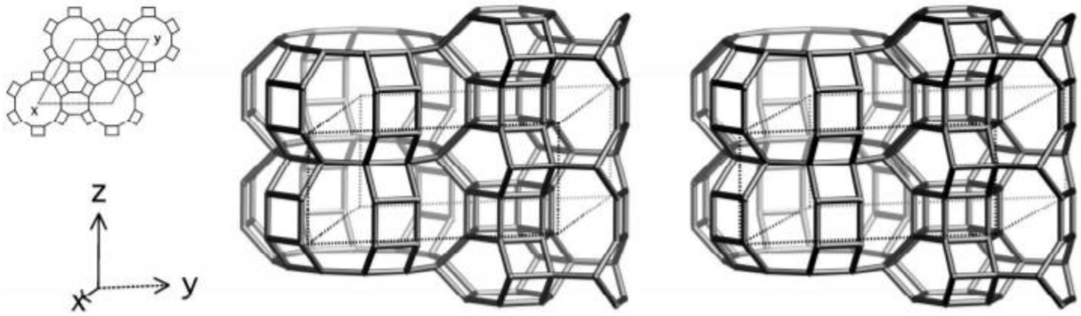

framework viewed normal to [001]

MFI

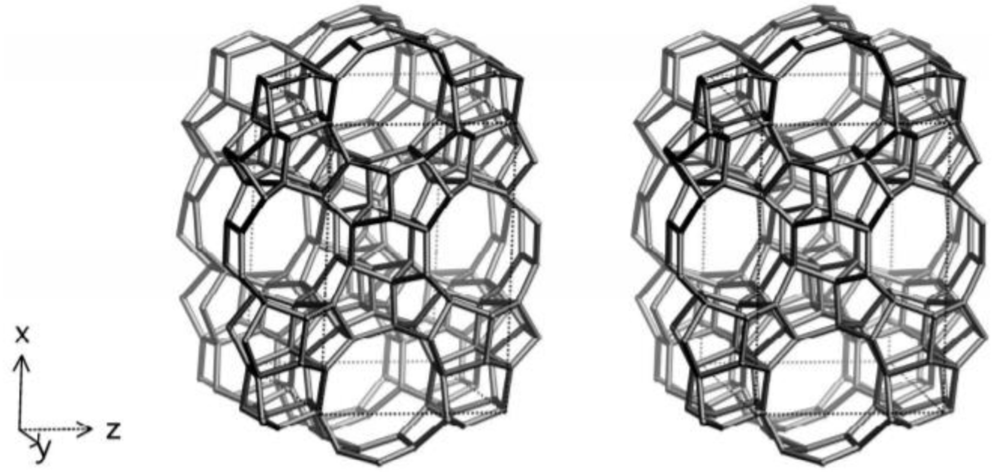

framework viewed along [010]

MOR

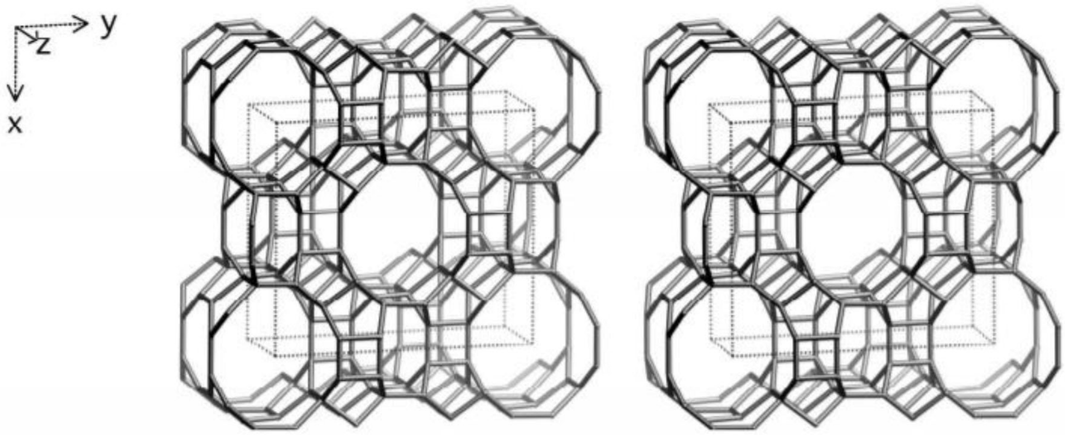

framework viewed along [001]

MW
W

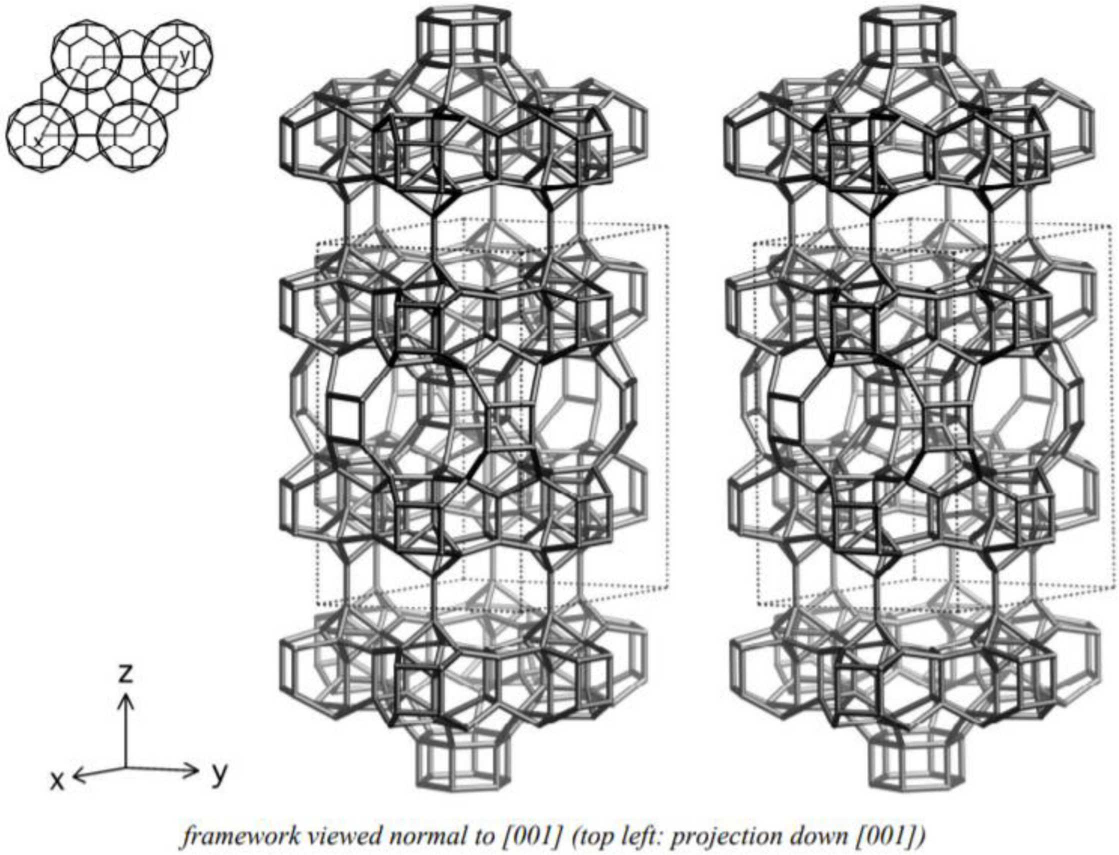

Figure S2. Framework types of selected zeolites. Zeolites X and Y mentioned in the manuscript have FAU framework type, zeolite A – LTA, zeolite ZSM5 – MFI, zeolite MCM-21 – MWW.

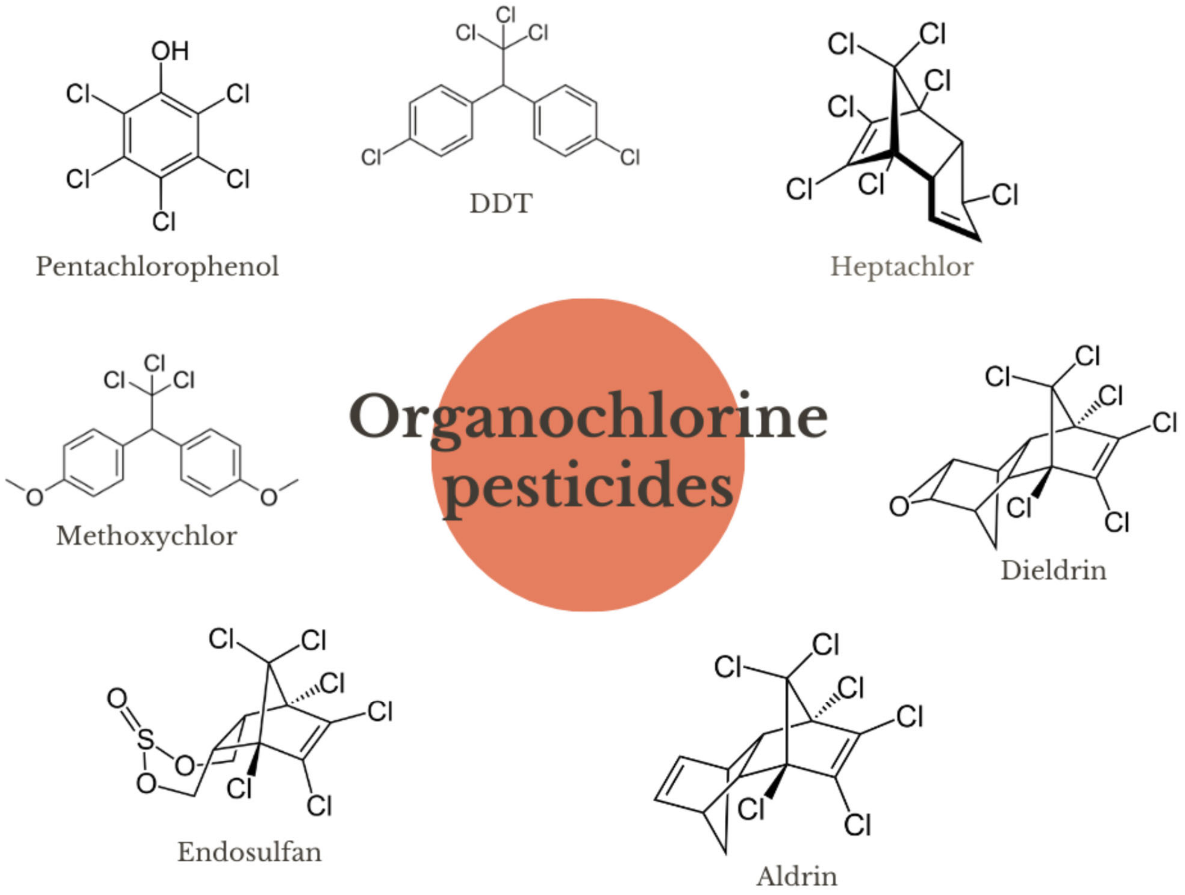

Figure S3. Structures of organochlorine pesticides.

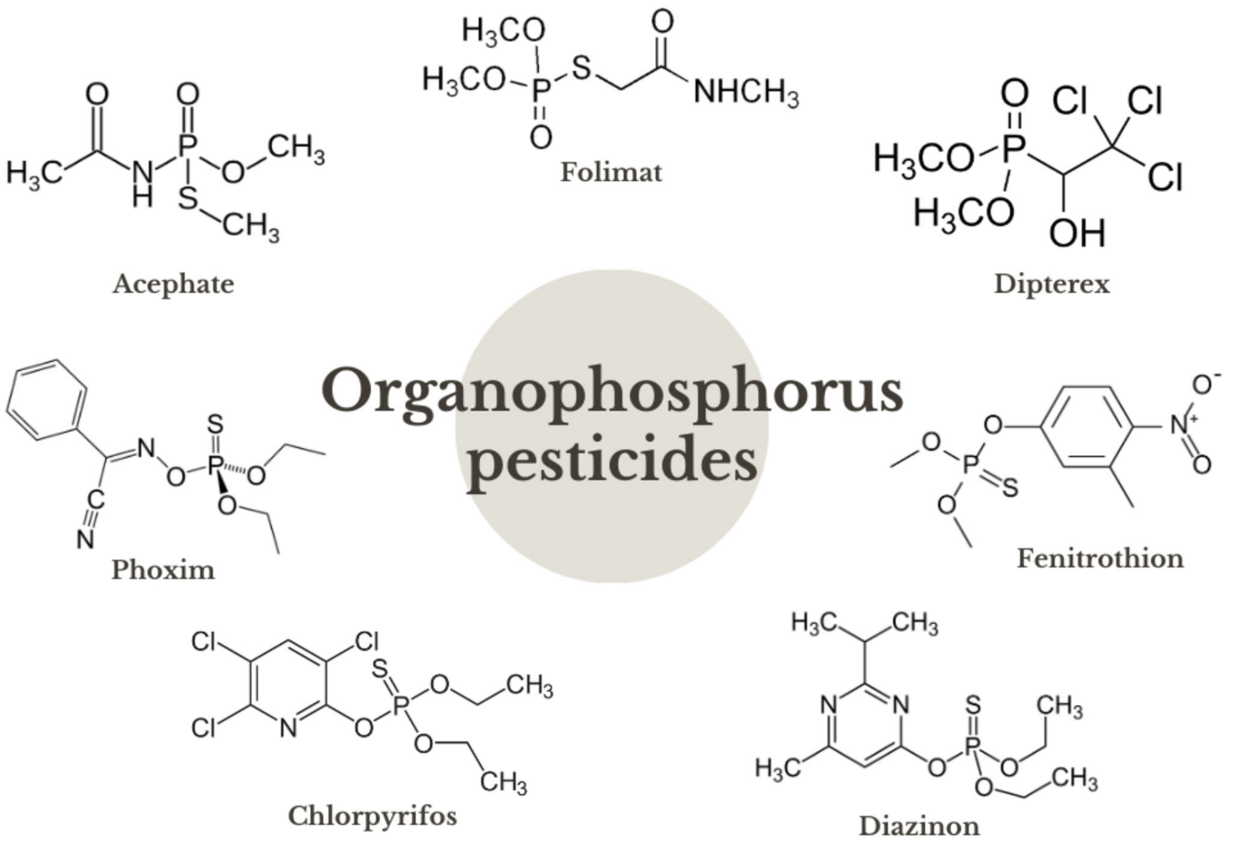

Figure S4. Structures of organophosphorus pesticides.

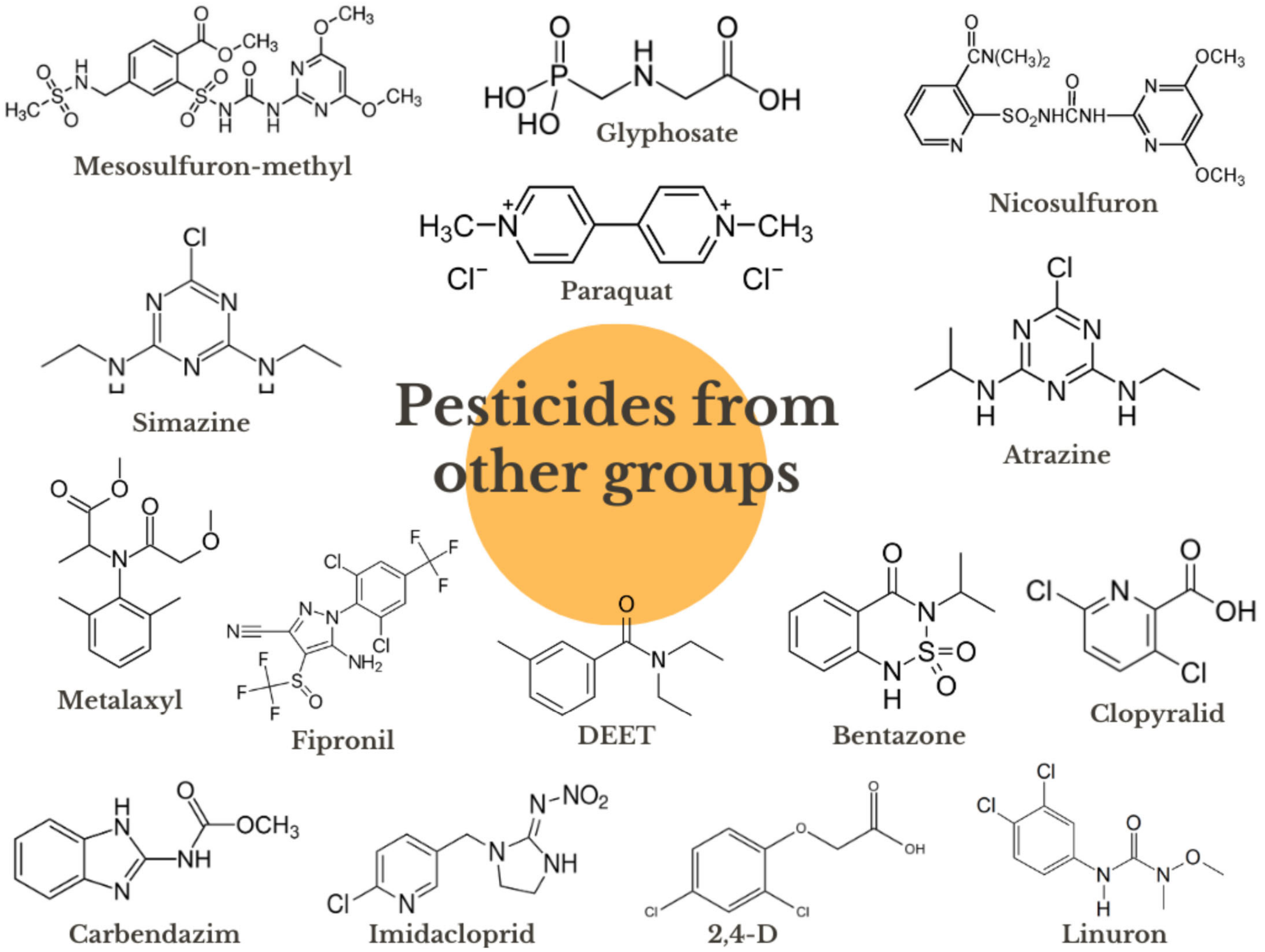

Figure S5. Structures of pesticides from other groups.

Table S1. Selected adsorption capacities and other parameters for the removal of organochlorine pesticides by mesoporous silica materials.

Adsorbate	Adsorbent	Adsorption (%)	Adsorption (mg/g)	Conditions: Conc. range¹ (mg/L)	Conditions: pH (-)	Conditions: Time (h)	Conditions: Temperature (°C)	Conditions: Adsorb. dosage² (g/L)	Reference
DDT	SBA-15	99	5.66*	8	-	12	-	1.4	[100]
	MCM-41	92	0.68*	1.03		36			
	MCM-48	89.5	0.66*						
	HMS	93.5	0.69*						
DDT	SBA-15	98	-	4	-	12	20	-	[99]
DDD		99	-						
DDE		96	-						
DDT	Fe-SBA-15	94–97	-						
DDD		98–99	-						
DDE		98	-						
Pentachlorophenol	SBA-15	5.8	5.79	10	6.5	12	25	4	[51]
	SA-SBA-15	32.8	3.28						
	ST-SBA-15	24.6	2.46						
Pentachlorophenol	(Na)Al-MCM-41	37.71*	33	700	6	4	30	8	[97]
	(K)Al-MCM-41	40*	35						
	(Cu)Al-MCM-41	53.71*	47						
	(Cr)Al-MCM-41	53.71*	47						
Heptachlor	HMS	52.56*	0.027	0.075	-	24	4	1.46	[98]
Endosulfan		85.51*	0.041	0.070					
Aldrin		60.65*	0.027	0.065					
Dieldrin		41.15*	0.031	0.110					
DDT		63.71*	0.024	0.055					
DDD		61.18*	0.044	0.105					
DDE		75.43*	0.031	0.060					
Methoxychlor		57.28*	0.051	0.130					
Heptachlor	CD-HMS-2%	66.27*	0.035	0.075				1.42	
Endosulfan		42.60*	0.021	0.070					
Aldrin		69.91*	0.032	0.065					
Dieldrin		54.22*	0.042	0.110					
DDT		64.55*	0.025	0.055					
DDD		71.68*	0.053	0.105					
DDE		82.83*	0.035	0.060					
Methoxychlor		68.82*	0.063	0.130					
DDT	HMS	37.77*	2.77	2.2	-	1	room	0.3	[101]
	Fe_3O_4@HMS-2	102.27*	7.5						

*—values calculated based on the data published in the cited articles; ¹—concentration range; ²—adsorbent dosage.

Table S2. Selected adsorption capacities and other parameters for the removal of organophosphorus pesticides by mesoporous silica materials.

Adsorbate	Adsorbent	Adsorption		Conditions					Reference
				Conc. range[1]	pH	Time	Temperature	Adsorb. dosage[2]	
		(%)	(mg/g)	(mg/L)	(-)	(h)	(°C)	(g/L)	
Acephate	SBA-15	0	0*	0.1	7	12	room	10	[104]
Folimat		3.9	0.00039						
Phoxim		2.0	0.0002*						
Chlorpyrifos		6.7	0.00067*						
Dipterex		4.9	0.00049*						
Acephate	SBA-15c	0	0*						
Folimat		6.4	0.00064*						
Phoxim		70.9	0.00709*						
Chlorpyrifos		84.6	0.00846						
Dipterex		7.4	0.00074*						
Acephate	SBA-15m	0	0*						
Folimat		8.4	0.00084*						
Phoxim		0	0*						
Chlorpyrifos		11.9	0.00119*						
Dipterex		10.9	0.00109*						
Acephate	MgO-SBA-15	6.1	0.00061*						
Folimat		16.6	0.00166*						
Phoxim		29.2	0.00292*						
Chlorpyrifos		22.7	0.00227*						
Dipterex		97.4	0.00974*						
Acephate	MCM-41	7.2	0.00072*						
Folimat		13.7	0.00137*						
Phoxim		20.1	0.00201*						
Chlorpyrifos		28.4	0.00284*						
Dipterex		19.2	0.00192*						
Acephate	MCM-41c	0	0*						
Folimat		6.3	0.00063*						
Phoxim		21.7	0.00217*						
Chlorpyrifos		47.5	0.00475*						
Dipterex		8.5	0.00085*						
Acephate	MCM-41m	0	0*						
Folimat		10.4	0.00104*						
Phoxim		2.6	0.00026*						
Chlorpyrifos		14.9	0.00149*						
Dipterex		13.7	0.00137*						

Acephate	MCM-48	0	0*						
Folimat		1.6	0.00016*						
Phoxim		4.4	0.00044*						
Chlorpyrifos		4.9	0.00049*						
Dipterex		10	0.0001*						
Diazinon	MCM-41	80	256*	320	-	24	room	1	[103]
Fenitrothion		100	56*	280				5	
Diazinon	MCM-48	75	240*	320				1	
Fenitrothion		67	37.52*	280				5	
Diazinon	MCM-41	55	9	50	9	1	25	3	[102]
	MPS-MCM-41	68	11						

*—values calculated based on the data published in the cited articles; [1]—concentration range; [2]—adsorbent dosage.

Table S3. Selected adsorption capacities and other parameters for the removal of different groups of pesticides by mesoporous silica materials.

Adsorbate	Adsorbent	Adsorption		Conditions					Reference
				Conc. range[1]	pH	Time	Temperature	Adsorb. dosage[2]	
		(%)	(mg/g)	(mg/L)	(-)	(h)	(°C)	(g/L)	
2,4-D	SBA-15/80	62.57*	117.32	150	3.13	4	-	0.8	[52]
	SBA-15/100	72.53*	136.00						
Mesosulfuron-methyl	SBA-15	20	1*	10	6	-	-	2	[107]
	SBA-15-SO3H	80	4*						
Glyphosate	SBA-15	14.8	0.05*	2	2.1	24	-	5.7	[109]
	Fe-SBA-15	100	0.35*						
	Fe-NH2-SBA-15	97	0.34*						
Carbendazim	SBA-15	1.1	0.00011*	0.1	7	12	room	10	[104]
Imidacloprid		1.2	0.00012*						
Carbendazim	SBA-15c	98.0	0.0098*						
Imidacloprid		23.0	0.0023*						
Carbendazim	SBA-15m	4.8	0.00048*						
Imidacloprid		7.4	0.00074*						
Carbendazim	MgO-SBA-15	8.7	0.00087*						
Imidacloprid		1.4	0.00014*						
Carbendazim	MCM-41	0	0*						
Imidacloprid		14.8	0.00148*						
Carbendazim	MCM-41c	82.9	0.00829*						
Imidacloprid		17.2	0.00172*						
Carbendazim	MCM-41m	4.2	0.00042*						
Imidacloprid		7.4	0.00074*						
Carbendazim	MCM-48	0	0*						
Imidacloprid		0	0*						
DEET	MCM-41	1*	5	1000	-	24	25	2	[118]
	MCM-41-S1	16*	80						
	MCM-41-S2	28*	140						
	MCM-41-Ph	12*	60						
DEET	HMS	1*	5						
	HMS-S2	23.6*	118						
Paraquat	MCM-41	7.29*	21	720	-	1	room	2.5	[115]
	25%Al-MCM-41	31.25*	70	560					[114]
Bentazone	MCM-41 (MS1)	60.8*	0.152	5	2.5	26	room	20	[119]
Avermectin	MCM-41	3.90*	78	10,000	-	24	35	5	[113]
	SA-MCM-41	2.90*	58						
	Mn-MCM-41	6.25*	125						

	Cu-MCM-41	7*	140						
	Zn-MCM-41	7.55*	151						
2,4-D	MCM-41	0*	0	440	4.5	6	25	2.5	[105]
	0.24APTES-MCM-41	75*	132						
Paraquat	HMS	54.08*	23.81	275.16	9.5	1	25	6.25	[116]

*—values calculated based on the data published in the cited articles; 1—concentration range; 2—adsorbent dosage.

Table S4. Selected adsorption capacities and other parameters for the removal organochlorine and organophosphorus pesticides by synthetic zeolites.

Adsorbate	Adsorbent	Adsorption		Conditions					Reference
				Conc. range[1]	pH	Time	Temperature	Adsorb. dosage[2]	
		(%)	(mg/g)	(mg/L)	(-)	(h)	(°C)	(g/L)	
α-endosulfan	HY(40)	-	775	30	-	1	room	-	[121]
	HBEA (Cal)	-	360						
	St700(3)	-	650						
Acephate	NaY	7.9	0.00079*	0.1	7	12	room	10	[104]
Folimat		3.4	0.00034*						
Phoxim		5.6	0.00056*						
Chlorpyrifos		11.0	0.0011*						
Dipterex		15.1	0.00151*						
Acephate	FeNaY (imp-3.5%)	7.6	0.00076*						
Folimat		17.9	0.00179*						
Phoxim		31.8	0.00318*						
Chlorpyrifos		31.4	0.00314*						
Dipterex		93.4	0.00934*						
Acephate	FeY (ex-8.6%)	20.6	0.00206*						
Folimat		18.3	0.00183*						
Phoxim		19.1	0.00191*						
Chlorpyrifos		19.5	0.00195*						
Dipterex		31.9	0.00319*						
Acephate	NTY	4.0	0.0004*						
Folimat		1.9	0.00019*						
Phoxim		10.7	0.00107*						
Chlorpyrifos		27.0	0.0027*						
Dipterex		13.3	0.00133*						
Acephate	MCM-22	8.0	0.0008*						
Folimat		3.9	0.00039*						
Phoxim		20.7	0.00207*						
Chlorpyrifos		30.8	0.00308*						
Dipterex		16.7	0.00167*						

*—values calculated based on the data published in the cited articles; [1]—concentration range; [2]—adsorbent dosage.

Table S5. Selected adsorption capacities and other parameters for the removal of different groups of pesticides by synthetic zeolites.

Adsorbate	Adsorbent	Adsorption		Conditions					Reference
				Conc. range¹	pH	Time	Temperature	Adsorb. dosage²	
		(%)	(mg/g)	(mg/L)	(-)	(h)	(°C)	(g/L)	
2,4-D	HY	3.2*							
	10HHY								
	10SHY								
	NaY				3				
	10HNaY			250		24	28	1	[106]
	10SNaY								
Paraquat	HY				11				
	30SHY								
	NaY								
	30SNaY								
Paraquat	NaY	64.24*	185	720	-	1	room	2.5	[115]
	NaBEA	42.36*	122						
Carbendazim	NaY	4.5	0.00045*	0.1	7	12	room	10	[104]
Imidacloprid		0	0*						
Carbendazim	FaNaY (imp-3.5%)	12.0	0.0012*						
Imidacloprid		7.9	0.00079*						
Carbendazim	FeY (ex-8.6%)	20.2	0.00202*						
Imidacloprid		21.5	0.00215*						
Carbendazim	NTY	74.9	0.00749*						
Imidacloprid		13.3	0.00133*						
Carbendazim	MCM-22#	100	0.01*						
Imidacloprid		1.9	0.00019*						
Paraquat	NAX	41.67*	120	720	-	1	room	2.5	[114]
Fipronil	H-ZSM-5	30	270*	900	2	2	25	1	[122]
	Ce25ZSM-5	79.6	716.4*						
	Ce30ZSM-5	76.7	690.3*						
Bentazone	beta (BEA)-1	69.6	1.39*	10	-	-	22	5	[120]
Clopyralid		9.1	0.18*						
Imidacloprid		98.8	1.98*						
Metalaxyl-m		42.0	0.84*						
Bentazone	Y (FAU)-7	100	2						
Clopyralid		10.8	0.22*						
Imidacloprid		99.7	1.99*						
Metalaxyl-m		99.3	1.99*						
Bentazone	4A (LTA)-11	0.2	0.004*						

Clopyralid		0	0*						
Imidacloprid		0	0*						
Metalaxyl-m		64.6	1.29*						
Bentazone	ZSM-5/silicalite-1 (MFI)-14	21.7	0.43*						
Clopyralid		3.9	0.08*						
Imidacloprid		86.1	1.72*						
Metalaxyl-m		36.9	0.74*						
Paraquat	H_LTL	10.4*	26	500	11	24	30	2	[117]
	K_LTL♯	66.8*	167						

*—values calculated based on the data published in the cited articles; [1]—concentration range; [2]—adsorbent dosage.

Table S6. Selected adsorption capacities and other parameters for the removal of triazine and urea pesticides by synthetic zeolites.

Adsorbate	Adsorbent	Adsorption		Conditions					Reference
				Conc. range¹	pH	Time	Temperature	Adsorb. dosage²	
		(%)	(mg/g)	(mg/L)	(-)	(h)	(°C)	(g/L)	
Simazine	HY	12.4*	2.48	2	6.5	24	25	0.1	[123,124]
Simazine	HY	12.51*	2.501	2	6.5	24	25	0.1	[125]
Atrazine	Y	35*	0.007	0.01	-	168	20	0.5	[126]
	ZSM-5	85*	0.017						
Atrazine	Y	3	0.06*	8	-	24	-	8	[127]#
	Y-10	33	0.66*						
	Y-100	90	1.80*						
	MY	2	0.04*						
	MY-10	53	1.06*						
	MY-100	94	1.88*						
Linuron	Y	32	0.64*						
	Y-10	74	1.48*						
	Y-100	100	2*						
	MY	4	0.08*						
	MY-10	78	1.56*						
	MY-100	99	1.98*						
Atrazine	A	48	480*	500	7	9	25	0.5	[128]
	X	54	540*						
Nicosulfuron	BEA	-	12	-	5	-	-	-	[130]
	BPW20	-	12						
	BPW20C#	-	15						
	BPW20U	-	26						
Nicosulfuron	BEA	34*	17	100	5	20	23	2	[131]
	PANI/BEA 1/1 H_2SO_4	48*	24						
	PANI/BEA 1/1 H_2O	50*	25						
	PANI/BEA 1/1 H_2SO_4 d.	26*	13						
	PANI/BEA 1/1 H_2O d.	22*	11						
Nicosulfuron	BEA	28*	14	100	5	20	23	2	[129]
	AgBEA	72*	36						
	BAgPW-TI10	32*	16						
	BAgPW-IE10	68*	34						
	BAgPW-PM10	18*	9						
Isoproturon	beta (BEA)-1	100	2*	10	-	-	22	5	[120]
	Y (FAU)-7	100	2*						
	4A (LTA)-11	0	0*						

	ZSM-5/silicalite-1 (MFI)-14	86.1	1.72*						
Mesosulfuron-methyl	HZSM-5	36*	3.6	20	6	72	25	2	[108]
	HZSM-5-60	41*	4.1						
	HZSM-5-60-Ac	44*	4.4						
	HMOR	0*	0						
	HMOR-70	29*	2.9						
	HMOR-70-Ac	41*	4.1						
Nicosulfuron	Z30	17.33*	6.5	75	5	24	23	2	[132]
	Z30K/PW-D	22.66*	8.5						
	Z30PW/K-C	28*	10.5						
	Z30PW/K-D	32*	12						
	Z30K/PW-C	76*	28.5						

*—values calculated based on the data published in the cited articles; [1]—concentration range; [2]—adsorbent dosage.

Table S7. Selected adsorption capacities and other parameters for the removal of glyphosate-based pesticides by synthetic zeolites.

Adsorbate	Adsorbent	Adsorption		Conditions					Reference
				Conc. range¹	pH	Time	Temperature	Adsorb. dosage²	
		(%)	(mg/g)	(mg/L)	(-)	(h)	(°C)	(g/L)	
Glyphosate	BEA	4.31*	34.5	4000	-	24	23	5	[110]
	BKPW-2	7.11*	56.9						
	BKPW-3	7.73*	61.8						
	BKPW-2C	11.53*	92.2						
	BKPW-3C	10.18*	81.4						
Glyphosate	HZSM-5	3.73*	14.9	4000	1.7	2	-	10	[111]
	PZ 1/1	6.70*	26.8						
	PZ 1/5	8.43*	33.7						
	PZ 1/10	4.63*	18.5						
	PZ 1/1 S	6.6*	26.4						
	PZ 1/1 d	15.48*	61.9						
	PZ 1/1 Sd	14.08*	56.3						
Glyphosate	4A	76*	38	100	3	2	-	2	[112]
	Cu-4A	80*	40						

*—values calculated based on the data published in the cited articles; ¹—concentration range; ²—adsorbent dosage.#
